# Scalp cooling versus standard cold-cap in preventing chemotherapy-induced alopecia in patients with localized breast cancer: ICELAND protocol, a randomized controlled trial and economic evaluation

**DOI:** 10.1371/journal.pone.0354676

**Published:** 2026-08-13

**Authors:** François Gernier, Justine Lequesne, Jean-Michel Grellard, Marie Fernette, Laetitia Gigan, Adeline Morel, Adrien Estienne, Hervé Castel, François Lahaye, Tiphaine Leroux, Rose-Marie Charles, Charlotte Dupont, Kevin Zarca, Nathalie Dereumaux, Anne-Cécile Rouanet, Isabelle Durand-Zaleski, Bénédicte Clarisse

**Affiliations:** 1 Clinical Research Department, Centre François Baclesse, Caen, France; 2 ANTICIPE (Interdisciplinary Research Unit for the Prevention and Treatment of Cancer), INSERM Unit 1086, Caen, France; 3 Medical Oncology Department, Centre François Baclesse, Caen France; 4 Breast pathway nurse, Centre François Baclesse, Caen, France; 5 Assistance Publique-Hôpitaux de Paris, DRCI-URC Eco Ile-de-France, Paris, France; 6 Université de Paris, Research Centre of Research Epidemiology and Statistics (CRESS-UMR1153), Inserm, Paris, France; 7 Health care manager, Centre Oscar Lambret, Lille, France; 8 Health care manager, Toulouse University Cancer Institute – Oncopole, Toulouse, France; Wuhu Hospital Affiliated to East China Normal University, CHINA

## Abstract

**Background:**

Chemotherapy-induced alopecia (CIA) remains one of the most distressing adverse effects for patients with localized breast cancer receiving anthracycline and taxane-based chemotherapy regimens. Although automated scalp cooling systems and standard non-automated cold-cap devices have demonstrated efficacy in reducing CIA, controlled trials with direct randomization comparing these two modalities of scalp cooling are lacking. This study aims to compare clinical efficacy, patient-reported outcomes, and medico-economic impact of a scalp cooling system (PAXMAN® PSCS 2) versus standard cold-cap therapy in preventing CIA.

**Methods/Design:**

ICELAND is a prospective, multicenter, open-label, randomized controlled phase III trial conducted in three French cancer centers. This study addresses female adult patients with histologically confirmed, non-metastatic breast cancer, scheduled to receive neoadjuvant or adjuvant chemotherapy with epirubicin-cyclophosphamide (3 or 4 cycles) followed by either docetaxel (3 cycles) or paclitaxel (9–12 weekly cycles) as per routine practice. A total of 196 patients will be 1:1 randomized to automated scalp cooling device (experimental arm) or standard non-automated cold-cap therapy (control arm), with stratification on chemotherapy regimen, menopausal status and centre. The primary endpoint is the proportion of patients experiencing no more than half hair loss (NCI-CTCAE v5.0 grade 0–1) from baseline to chemotherapy completion, as assessed by blinded central review of standardized photographs. Secondary endpoints include: degree of CIA before taxane initiation; hair regrowth 6 months after the end of chemotherapy; patient adherence and comfort for each scalp cooling modality; body image (BIS); quality of life (EORTC QLQ-C30/BR23); anxiety/depression (HADS); satisfaction with care (OUTPATSAT-35-CT); return to work; nursing time associated with device use; and cost-utility in QALYs.

**Discussion:**

This study will provide the first high-quality randomized evidence directly comparing scalp cooling and standard cold-cap use for CIA prevention in localized breast cancer. The results will inform both clinical practice and health policy by integrating clinical efficacy, patient-reported outcomes, and medico-economic considerations.

**Trial registration:**

ClinicalTrials.gov NCT06011525

## Introduction

Localized breast cancer accounts for over 60,000 new cases yearly in France. Approximately one quarter of these patients require anthracycline- and taxane-based chemotherapy as part of their neoadjuvant or adjuvant treatment regimen (typically three to four cycles of epirubicin–cyclophosphamide [EC] followed by either three cycles of docetaxel or 9–12 weekly cycles of paclitaxel). These regimens are highly hair-loss inducing and cause chemotherapy-induced alopecia (CIA) in the vast majority of patients in the absence of preventive measures. CIA occurs in most of cases within the first two weeks after the first dose of chemotherapy. It is usually transient but persistent alopecia is reported in up to 10% of cases [1]. Hair regrowth usually begins 4–6 weeks after chemotherapy completion but may be delayed or of suboptimal quality, especially following anthracycline or taxane exposure [2]. For patients, hair loss is often perceived as a visible hallmark of cancer, and sometimes the only outward sign of illness. CIA is thus one of the most distressing chemotherapy-related adverse effects, with substantial psychosocial repercussions, including impaired body image and decreased psychosocial well-being and quality of life [3].

From the last decades, scalp hypothermia has been widely used to prevent and decrease CIA. Nowadays, scalp cooling can be achieved using either cold-cap systems, or the more recent scalp cooling machines: they are currently the only evidence-supported interventions [4,5]. Standard cold-cap systems consist in glycerin-based gel caps frozen and stored at temperature around −18°C to −25°C, applied directly to the scalp during a time overlapping chemotherapy administration. These caps allow generating surface temperatures around –15°C but have to be replaced every 30–45 minutes to maintain efficacy throughout the chemotherapy course. Automated scalp cooling systems (e.g., PAXMAN® PSCS 2) include a computer-controlled refrigeration unit that circulates coolant at –4°C through a fitted cap, maintaining continuous and stable temperature cooling scalp throughout chemotherapy infusion and post-infusion. These two modalities of scalp cooling for CIA prophylaxis are suitable for patients harbouring solid tumors with the exception of central nervous tumors, and contraindicated for patients with brain or scalp metastases, or with some hematological malignancies. Safety evaluations have shown no increased risk of scalp metastases with these techniques [6–10].

Mechanistically, these devices exert a compressive and cooling effect on the scalp, leading to vasoconstriction, which reduces local blood flow and thereby limits delivery of cytotoxic agents administered intravenously to hair follicles. Additionally, scalp cooling may reduce (i) mitotic activity in follicular keratinocytes, making them less susceptible to chemotherapy-induced damage, (ii) as well as intra-follicular metabolic rate [6]. Preservation of hair follicles may also result in faster and more homogeneous hair regrowth, an effect of particular interest in taxane-based regimens, which can impair regrowth quality [2].

In routine practice, scalp cooling use in outpatient oncology care may suffer from the prioritization of therapeutic care and the relegation of supportive care to a secondary position [11,12]. It indeed implies organizational challenges as it requires additional nursing time, extended chair time for pre- and post-infusion cooling, infrastructure investment (especially for automated systems), and dedicated storage space with freezers for gel caps. Hair loss prevention with scalp hypothermia may also be limited depending on mixed perceptions among healthcare professionals, stemming from concerns over tolerability (cold discomfort, headaches) and inconsistent efficacy.

To date, no randomized controlled trials have directly compared automated scalp cooling with standard non-automated cold-cap therapy. Most randomized studies already conducted compared scalp cooling with no intervention, and few have included patients receiving anthracycline-based regimens [4,5,13]. As concerning efficacy, breast cancer patients using the standard cooling cap during standard chemotherapy (namely, 3–4 EC followed by 3 Docetaxel courses, or 3–4 EC followed by 9–12 Paclitaxel courses) retained at least 50% of their hair in 20% to 36% of cases [14,15]. The scalp-cooling automated technique appears to be more effective in this indication, with up to 78% of patients retaining at least 50% of their hair after treatment with anthracyclines and taxanes [16,17]. In light of these results, and considering the ability of automated scalp cooling technique to maintain continuous and stable scalp temperature during intervention, we hypothesize that automated scalp cooling (PAXMAN® PSCS 2) will demonstrate higher efficacy in preventing CIA compared to standard cold-cap therapy in women with localized breast cancer receiving anthracycline and taxane-based chemotherapy, while also improving patient comfort, body image, quality of life, and cost-effectiveness.

## Methods

**Fig 1:**
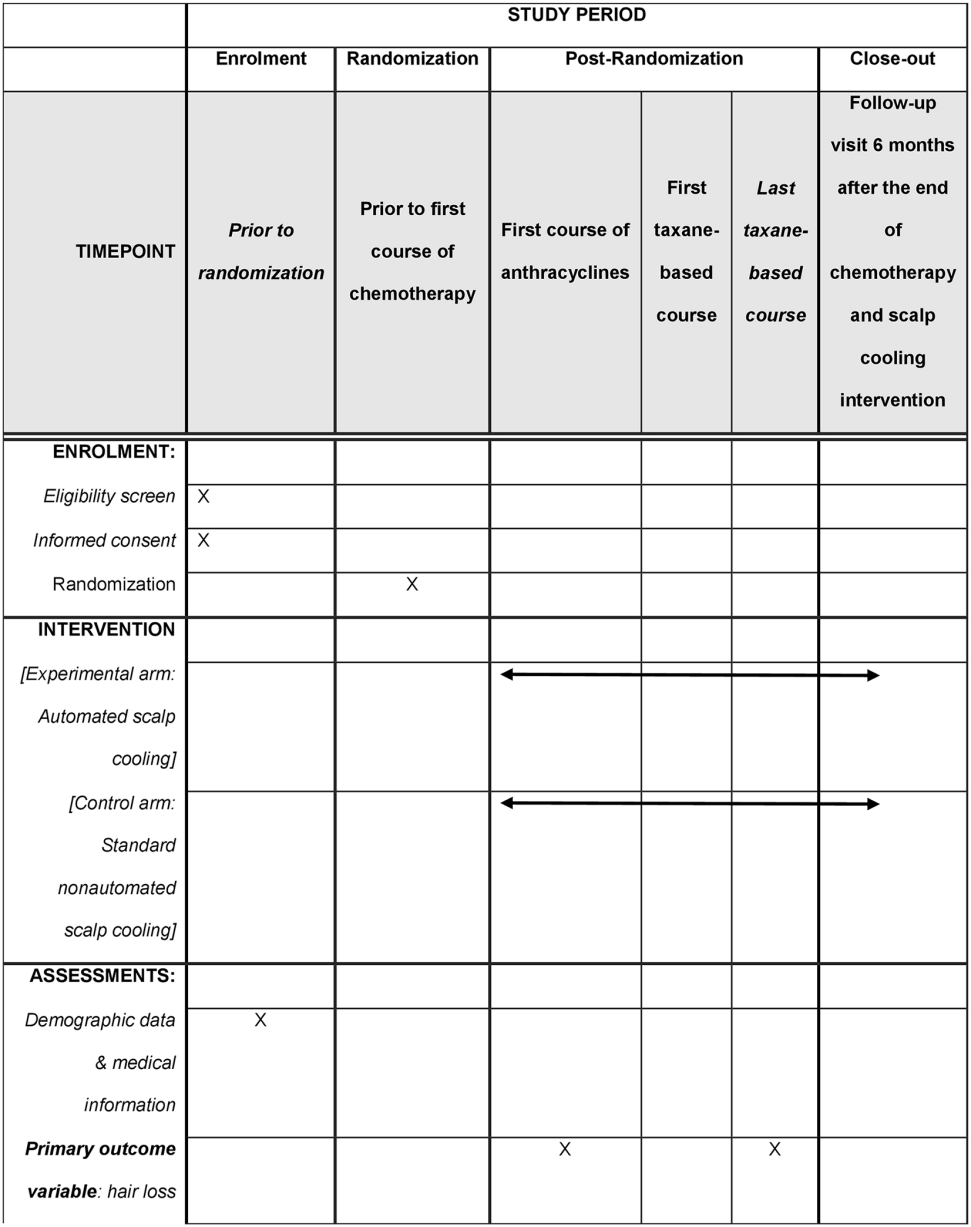
SPIRIT schedule of enrolment, interventions and assessments.

The ICELAND study is a prospective, randomized, multicenter, open-label, controlled phase III trial (https://www.clinicaltrials.gov/study/NCT06011525) addressing patients with an indication of a neoadjuvant or adjuvant chemotherapy treatment including anthracyclines and taxanes for a localized breast cancer. It compares two modalities of scalp cooling (automated versus standard nonautomated devices) for prevention of CIA. The study flow-chart is illustrated in **Fig. 1**.

The ICELAND protocol and the present manuscript have been written in accordance with the Standard Protocol Items: Recommendations for Interventional Trials (SPIRIT). The study has received ethical approval from the *Comité de Protection des Personnes Nord Ouest I* in August 2023 (N° IRD-RCB: 2023-A00769-36). The French Health Regulatory Authority (ANSM) validated the compliance of this clinical investigation with the EU Medical Device Regulation (MDR 2017/745, Article 82). All patients will be proposed to participate by nurses or medical oncologists and will provide their informed written consent before any study-related procedure. Confidentiality of participant data will be maintained in accordance with the French Data Protection Act and the General Data Protection Regulation (GDPR; EU 2016/679). This study will be conducted in accordance with the relevant guidelines and regulations (Declaration of Helsinki).

### Primary objective

The primary objective is to compare the efficacy of scalp cooling modality (automated scalp cooling versus standard cold caps) in terms of hair loss prevention among patients with localized breast cancer receiving anthracycline- and taxane-based regimens. The primary endpoint is the proportion of patients with up to 50% hair loss (NCI CTCAE v5.0 grade 0–1) from baseline to end of chemotherapy, assessed by blinded central review of standardized scalp photographs performed at inclusion and last course of chemotherapy.

### Secondary objectives

Secondary endpoints will be used to assess and compare between both arms:

- The severity of CIA prior to first taxane cycle, and 6 months after the end of chemotherapy (patient self-assessment and oncologist assessment).
- The quality of regrowth 6 months after the end of chemotherapy (patient self-assessment and oncologist assessment).
- The patient adherence to the modality of scalp cooling and reasons for discontinuation.
- The comfort level through a 4-point Likert scale on several parameters (comfort with regards to the scalp cooling device, cold sensation, and duration of scalp cooling) after first use, after the first taxane cycle and last use of the scalp cooling modality.
- Body image (using the Body Image Scale [18]).
- Quality of life (using the EORTC QLQ-C30 [19] and QLQ-BR23 [20] self-questionnaires).
- Anxiety/depression (using the Hospital Anxiety and Depression Scale, HADS [21]).
- Satisfaction with care, using the OUTPATSAT-35 CT self-questionnaire [22] at the last course of chemotherapy.
- Condition and time to return to work and/or normal daily activities.
- Nursing time per session for device fitting, use, and cleaning.
- Cost-utility (QALY) of automated scalp cooling versus standard cold caps.

### Study population

Eligibility criteria are listed in Table 1. The ICELAND study addresses patients with an indication for a neoadjuvant or adjuvant chemotherapy with an anthracycline and taxane-based regimen for a localized breast cancer.

**Table 1:** Eligibility criteria in the ICELAND study.

Inclusion criteria	Non-inclusion criteria
• Female patients ≥18 years. • Histologically confirmed localized, non-metastatic breast cancer. • Indication for neoadjuvant or adjuvant anthracycline and taxane-based chemotherapy (3–4 EC cycles followed by either 3 docetaxel cycles or 9–12 weekly paclitaxel cycles). • Willingness to undergo scalp photography for CIA assessment. • Proficiency in French language. • Affiliation with the French social security system. • Provision of written informed consent.	• Pre-existing alopecia. • History of cervical pain, migraine, cerebrovascular accident, hyperthyroidism, Raynaud’s syndrome, cryoglobulinemia, cold agglutinin disease, cold-induced post-traumatic dystrophy, or cryofibrinogenemia. • History of scalp metastases or hematological malignancy. • Prior chemotherapy. • Scalp skin disorders or contraindication to scalp cooling. • Pregnancy or lactation. • Planned cranial radiotherapy. • Participation in another clinical trial.

### Study site

The study is conducted across three French comprehensive cancer centers (https://www.clinicaltrials.gov/study/NCT06011525#contacts-and-locations).

### Study experimental plan

**Fig 2:**
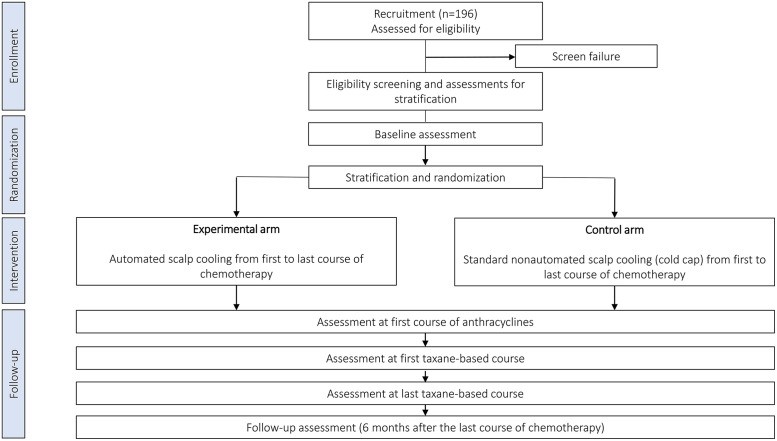
ICELAND study diagram.

The study will be proposed to eligible patients prior to chemotherapy initiation by nurses or medical oncologists (Fig 2). Patients with informed signed consent will be 1:1 randomized to receive either automated scalp cooling (experimental arm) or standard non-automated cold-cap device (control arm) during chemotherapy.

Randomization will be performed using a centralized web-based system (Ennov Clinical®) within 8 days of inclusion, through a minimization algorithm incorporating a random factor of 0.9, with stratification on chemotherapy regimen (docetaxel vs paclitaxel), study center and menopausal status.

### Scalp-cooling intervention

For patients in both arms, the scalp cooling device will be applied by a nurse, according to the device manual, during each course of chemotherapy, with application starting at least 15 minutes before the infusion begins, kept in place all over the perfusion duration and left at least 20 minutes after the infusion ends. According to physician decision, prophylactic paracetamol (1 gram *per os*) may be administered before each course to prevent from the risk of headache induced by scalp cooling.

The intervention will last up to the last course of chemotherapy, unless the patient prematurely withdraws from the study.

Participation to this study does not modify the therapeutic care of enrolled patients. Data on chemotherapy regimen administered, including possible dose adaptation and/or administration delay will be collected.

- *Automated scalp cooling (experimental arm)*

For patients in the experimental arm, the Paxman Scalp Cooling System 2 (PCS2, Paxman Coolers Limited, Huddersfield, United Kingdom) will be used. This is a freestanding, electrically powered, mobile refrigeration unit consisting in circulating a liquid coolant at a preset temperature (−4°C in this study) and flow rate through a cooling cap attached to the top of the patient’s head. Hair preparation includes dampening hair with warm water, applying a small amount of conditioner, combing hair away from face to expose hairline, and placing a mesh headband under the ears and across the forehead. Cap is fitted according to manufacturer’s guidelines, ensuring snug contact over scalp surface. It is applied 30 min before infusion (45 minutes for thick or curly hair), maintained continuously for the entire chemotherapy administration, and up to 120 minutes after EC cycles; 20 minutes after paclitaxel or docetaxel. All nurses will be trained before the first inclusion, using a standardized fitting procedure to ensure optimal cap size selection and scalp contact, as improper fitting is known to reduce cooling efficacy.

- *Non-automated scalp cooling (control arm)*

For patients in the control arm, glycerin-based gel caps stored at ≤–20°C will be used. Scalp preparation includes hair moistening with cold water prior to cap placement and cotton pads application over ears and forehead to prevent frost injury. Cold caps will be applied at least 15 minutes before infusion start, with compression bandage: applied over the cap to enhance vasoconstriction, replaced every 45 minutes to maintain cooling, and kept 20 minutes after infusion completion.

### Study assessments

All enrolled patients in both arms will be assessed at baseline before randomization, during chemotherapy and 6 months after the end of chemotherapy, at the time of the first standard oncological evaluation (Table 2).

**Table 2:** Study flow-chart.

	Study information & consent	Inclusion & baseline evaluation	Randomization (ratio 1:1): Automated scalp cooling (experimental arm) *versus* Standard non-automated scalp cooling (control arm)	First course of anthracyclines (EC)	First taxane-based course *(Docetaxel or Paclitaxel)*	Last taxane-based course *(Docetaxel or Paclitaxel)*	Follow-up visit 6 months after the end of chemotherapy
**Patient information and reflection period** **Informed written consent**	**•** **•**						
**Inclusion**		**•**					
**Clinical exam** (cancer characteristics, medical antecedents, signs and symptoms…)		**•**					
**Quality of life** (EORTC QLQ-C30 & QLQ BR23)		**•**			**•**	**•**	•
**Anxiety/Depression** (HADS)		**•**			**•**	**•**	**•**
**Body Image index** (BIS)		**•**			**•**	**•**	**•**
**Safety and comfort related to scalp cooling device** (4-point Likert scale)				**•**	**•**	**•**	
**Scalp photograph realized by a health provider**				**•**		**•**	
**Visual hair assessment** - by the patient **-** by the nurse and/or medical oncologist					**•**	**•**	**•**
**Return-to-work and/or daily activities**							**•**
**Satisfaction with care** (OUT-PATSAT35-CT)						**•**	
**Return-to-work and/or daily activities conditions**							**•**
**Out-of-pocket costs for wigs and CIA-related care**							**•**

Evaluations will include:

- A clinical exam, with collection of demographic data, medical history, tumor characteristics, and planned chemotherapy regimen, performed prior to randomization.

- The completion of self-questionnaires prior to randomization, at first taxane-based course and last chemotherapy infusion, to assess:

- Health-related quality of life, using the EORTC QLQ-C30 [19] questionnaire and the QLQ BR23 module for breast cancer [20]
- The level of anxiety and depression, using the HADS [21]
- The Body image, through the BIS [18]

- Hair loss assessment:

- Through a standardized scalp photograph realized by a health provider at baseline (before the first infusion) and at the end of chemotherapy. Standardized photograph (vertex) will be taken as far as possible at a fixed distance under consistent lighting conditions, following a dedicated photographic manual. Two independent, blinded reviewers will grade hair loss according to NCI‑CTCAE v5.0; if necessary, discrepancies will be resolved by consensus or a third reviewer.
- Visual evaluation at each infusion by the nurse and/or the medical oncologist, and the patient herself, as well as at 6 months of follow-up.
- The quality of hair regrowth visually assessed by the nurse and/or the medical oncologist, and the patient at 6 months of follow-up.

- The device comfort level reported by the patient through a 4-point Likert scale on several parameters (comfort with regards to the scalp cooling device, cold sensation, and duration of scalp cooling) after first use, after the first taxane cycle and last course of chemotherapy

- Nurse-report of time spent for scalp cooling device use at each infusion, with regards to time for application, change, remove, and disinfection.

- Patient-report on satisfaction with care, using the OUTPATSAT-35 CT questionnaire [22] at the last course of chemotherapy.

- At 6 months of follow-up, patients will report their return-to-work and/or daily activities conditions, as well as the out-of-pocket costs for wigs and CIA-related care

### Medico-economic study

A within-study health economic analysis will be conducted from the perspective of the healthcare system with a 12-month time horizon (chemotherapy treatment duration and 6 months of follow-up). The analysis population will include enrolled patients, analyzed using an intention-to-treat (ITT) approach.

A cost-utility analysis will be performed, in which health outcomes will be measured in survival and valued in QALYs (Quality adjusted life years), assuming that automated scalp cooling will measurably improve patients’ quality of life over a short time horizon.

The different dimensions of the EORTC QLQ C30 questionnaire, measured over the patients’ participation, will be mapped to the dimensions of the EQ5D-5L questionnaire using the coefficients of an ordered logistic regression [23]. The French weightings to the dimensions of the EQ5D-5L questionnaire will then be applied to obtain utilities [24] in each arm. The utility will be linearly interpolated between each time point and QALYs will be obtained by calculating the area under the utility curve as a function of time (dQALY/dt = S(t)U(t)).

The cost estimate will include all healthcare costs related to the management and treatment of CIA throughout the study. Only direct costs will be considered in the analysis, in accordance with the recommendations of the French National Authority for Health (HAS). Costs related to the intervention will be collected, including:

- Purchase cost of the device, including depreciation over the device lifespan, as well as electricity consumption associated with each modality of scalp cooling (freezer for cold caps freezing, automated scalp cooling device)
- Human resource costs, based on involved nurse reports regarding amount of work assigned and time spent for scalp cooling device use at each infusion, including time for preparing for device placement, monitoring, device change and removal, and cleaning, over the period from the patients’ arrival to their discharge from outpatient hospital, through micro-costing.

Assuming that hospitalizations are unlikely to occur due to the use of the device, as its benefit lies in comfort and quality of life, data on hospital care consumption will not be collected.

Human resources will be valued at the total annual cost of the position, and the cost of the device will be valued at the hospital’s purchasing department.

The cost-effectiveness ratios (ICERs), defined as (cost of usual nonautomated care – cost of the automated scalp cooling strategy)/ (QALY with usual nonautomated care – QALY with automated scalp cooling), will be calculated.

Costs and utility will be presented as means with 95% confidence intervals. Lastly, the robustness of the results will be assessed using a deterministic sensitivity analysis to account for the uncertainty of the parameters studied by varying their values. A bootstrapped analysis of the joint distribution of costs and QALYs will also be performed. An acceptability curve will be presented to interpret the efficiency results according to the chosen threshold ratio.

### Statistical considerations

#### Sample size.

The trial is powered to detect a clinically significant difference in the proportion of patients with ≤50% hair loss (CTCAE v5.0 grade 0–1) between the two intervention arms at the end of chemotherapy. Assuming a success rate of *50%* in the automated scalp cooling arm [14,15] and *30%* in the standard cold-cap arm [16], with α = 0.05 (two-sided) and 80% power, a total sample size of 196 patients (98 per arm) is required, accounting for a 5% dropout rate.

#### Statistical analysis.

The primary endpoint will be analysed using a logistic regression model adjusted for stratification factors (chemotherapy regimen, menopausal status, center). Treatment effects will be reported as odds ratios with 95% confidence intervals. An unadjusted chi-squared test will then be performed as a supportive analysis.

Analysis will be conducted with the intent to treat population, defined as all randomized patients according to their assigned treatment arm, whatever their adherence to device wearing. Analyses in the sample of patients completing the intervention as planned without major deviations (per-protocol) will be conducted as sensitivity analyses.

Ordinal and continuous outcomes (CIA grade, comfort scores, questionnaire scores) will be compared using non-parametric tests (Wilcoxon rank-sum) or linear mixed models for repeated measures. Adherence and device discontinuation rates will be summarized descriptively and compared using chi-squared tests.

### Quality control

#### Training of teams in participating centers.

To ensure harmonization of practices across all investigator centers, a comprehensive procedures manual will be prepared and approved by each center prior to the start of the study. Study staff at each center will be trained on the eligibility screening, consent process, modalities for use of scalp cooling devices, questionnaires, and scalp photographs.

#### Protocol deviations.

A standard protocol deviations form will be used to track any deviations. The study staff will maintain the protocol deviations log. Study staff will also record deviations identified during the data audit and reconcile errors.

#### Data monitoring and management.

Study staff will monitor data collection and protocol deviations. A data monitoring committee will not be necessary since the study is considered to be at minimal risk.

A Web-Based Data Capture (WBDC) system will be used for randomization, data collection and query handling. The investigator will ensure that data are recorded on the eCRFs as specified in the trial protocol and in accordance with the instructions provided. The investigator ensures the accuracy, completeness, and timeliness of the data recorded as well as of the provision of answers to data queries according to the Clinical Study Agreement. The investigator will be in charge of signature of the completed eCRFs. A copy of the completed eCRFs will be archived at the study site.

### Dissemination

Study results will be published in international peer-reviewed scientific journals and presented at relevant scientific conferences.

### Status and timeline of the study

This study is registered on ClinicalTrials.gov, identifier NCT06011525. First inclusion was realized on September 8, 2023. Recruitment is due to finish on September 8, 2027, and patients’ follow up complete on September 8, 2028. Final results are expected for 2029.

## Discussion

CIA remains a major quality-of-life concern for patients undergoing cytotoxic treatment for breast cancer. While automated scalp cooling and standard non-automated cold-cap therapy have both separately demonstrated efficacy in mitigating CIA, current evidence is predominantly derived from studies comparing each intervention to no prevention. In addition, most of conducted studies have not considered anthracycline-based regimens. To date, no randomized controlled trial has directly compared these two strategies of scalp cooling under identical clinical conditions for management of patients receiving a chemotherapy.

The ICELAND trial is designed to fill this evidence gap by comparing the clinical efficacy, patient experience, and cost-effectiveness of automated scalp cooling (PAXMAN® PSCS2) versus standard non-automated cold-cap therapy in a homogeneous population of women with localized breast cancer receiving anthracycline- and taxane-based chemotherapy. By integrating both objective assessments (photographic evaluation of hair preservation) and patient-reported outcomes (quality of life, body image, comfort with regards to device use, and satisfaction of care), this study adopts a comprehensive evaluation framework. The comparative randomized design with stratification factors allows robust and generalizable conclusions; however, some retrospective stratified analysis could be performed to mitigate results and consider potential allocation foreknowledge, as recommended by Hills et al [25].

From a clinical perspective, automated scalp cooling may offer more stable and sustained temperature control compared with cold-cap therapy, potentially improving follicular protection and hair preservation rates. Conversely, non-automated cold-cap therapy is widely available, less costly in terms of capital investment, and does not require dedicated equipment beyond gel caps and freezers. The balance between effectiveness, tolerability, and resource use will be critical for guiding adoption in routine oncology practice.

From a health economics standpoint, the ICELAND study incorporates a cost-utility analysis that will quantify the economic value of each technique, taking into account direct medical costs, non-medical costs, and productivity losses. This is particularly relevant in the context of limited healthcare resources and the need to optimize supportive care services without compromising treatment outcomes. It should be noticed that data related to return-to-work and out-of pocket cost will be assessed as study secondary endpoints distinct from the cost analysis, thus providing additional insight in terms of health economics.

If the study confirms superior clinical and economic performance of one technique over the other, it could influence institutional purchasing decisions, national supportive care guidelines, and reimbursement policies for CIA prevention in breast cancer patients.

## Conclusion

The ICELAND study will provide the first high-quality, direct randomized evidence comparing automated and standard non-automated scalp cooling devices for CIA prevention in women treated for a localized breast cancer. Its comprehensive design—integrating objective clinical outcomes, patient-reported measures, and economic evaluation—will generate actionable insights for clinicians, patients, and healthcare policymakers. Ultimately, the study seeks to optimize the delivery of CIA prevention strategies that improve patient well-being and are sustainable in diverse healthcare settings.

## Supporting information

S1 File SPIRIT checklist. (DOCX)

S2 File Copy of the ICELAND study protocol, version 5.1, dated from 2025/NOV/17. (PDF)
