# Supplementary material for: Scalp cooling versus standard cold-cap in preventing chemotherapy-induced alopecia in patients with localized breast cancer: ICELAND protocol, a randomized controlled trial and economic evaluation
Source: PLoS One. 2026 Aug 13;21(8):e0354676. doi: 10.1371/journal.pone.0354676 (PMC13472393; doi:10.1371/journal.pone.0354676)
Supplement: Supplementary file 1 [file pone.0354676.s002.pdf]

ICELAND TRIAL

Prevention of alopecIa in patients with localized breast CancEr using scalp cooLing vs cooling cAp: a raNDomized controlled trial with medical-economic evaluation

N°ID-RCB: 2023-A00769-36
Version No.5.1 dated from 11/17/2025

This trial is supported by the French Solidarity and Health Ministry, within a Hospital program for nursing and paramedical research grant (Programme hospitalier de recherche infirmière et paramédicale, PHRIP 21-0015).

REGULATORY CLASSIFICATION: Clinical investigation on a medical device with CE marking (Art. 82 RDM)

**SPONSOR**	**Centre François Baclesse**<br>3 avenue du Général Harris<br>14076 CAEN cedex 5, FR<br>Tél. : 33 2 31 45 50 50 – Fax : 33 2 31 45 51 58

**COORDONNATOR INVESTIGATOR**	**Rose-Marie CHARLES**<br>Nurse, breast care pathway<br>Centre François Baclesse<br>3 avenue du Général Harris<br>14076 CAEN cedex 5, France<br>rm.charles@baclesse.unicancer.fr<br>Tél. : 02 31 45 86 48

**COMPETENT AUTHORITY**	Ethics Committee:<br>CPP Nord-Ouest I	Date of approval: 2020-08-03<br>Amdt 1 approved: 2024-06-17<br>Amdt 2 approved: 2024-11-20<br>Amdt 3 approved: 2025-07-08

CONFIDENTIALITY STATEMENT

The information contained in this document is the property of Centre François Baclesse and therefore is provided to you in confidence for review by you, your team, an applicable Ethics Committee/Institutional Review and regulatory authorities. It is understood that the information will not be disclosed to third parties without prior written approval from Centre François Baclesse, except to the extent necessary to obtain informed consent from those persons to whom the medication may be administered.

PEOPLE INVOLVED IN THE PREPARATION AND THE CONDUCT OF THE PROTOCOL

COORDINATING INVESTIGATOR	
**COORDINATING INVESTIGATOR**<br>Rose-Marie CHARLES<br>*Nurse Breast Care Pathway*	**Centre François Baclesse – CAEN, France**<br>rm.charles@baclesse.unicancer.fr<br>Tel : +33 (0)2 31 45 86 48
**ASSOCIATE INVESTIGATION CENTER**<br>Nathalie DEREUMAUX<br>*Healthcare manager*<br><br>Sophie COPPENS<br>*Pole Manager, Day Hospital/Outpatient Care Sector*<br><br>Anne-Cécile ROUANET<br>*Healthcare manager*	**Centre Oscar Lambret – LILLE, France**<br>Tél : 03 20 29 59 59<br>N-Dereumaux@o-lambret.fr<br><br>s-coppens@o-lambret.fr<br><br>***IUCT – ONCOPOLE – TOULOUSE, France***<br>Rouanet.Anne-Cecile@iuct-oncopole.fr
**CO-COORDINATING INVESTIGATOR**<br>Charlotte DUPONT<br>*Nurse _ Project Manager*<br><br>Dr Adeline MOREL<br>*Medical oncologist*	**Centre François Baclesse – CAEN, France**<br>c.dupont@baclesse.unicancer.fr<br>Tél : 02 31 45 50 50, poste 56 94<br><br>a.morel@baclesse.unicancer.fr
**CLINICAL RESEARCH**	
**Unit Leader**<br>Bénédicte CLARISSE<br><br>**Project Manager**<br>Jean-Michel GRELLARD<br><br>**Methodologist**<br>Justine LEQUESNE	**Centre François Baclesse – CAEN, France**<br>Tel : +33 (0)2 31 45 50 02<br>Fax : +33 (0)2 31 45 51 58<br><br>b.clarisse@baclesse.unicancer.fr<br><br>jm.grellard@baclesse.unicancer.fr<br><br>j.lequesne@baclesse.unicancer.fr
**HEALTHCARE RESEARCH COORDINATOR**<br>François GERNIER, *Healthcare manager*	**Centre François Baclesse – CAEN, France**<br>f.gernier@baclesse.unicancer.fr<br>Tel : 02 31 45 40 43

**MEDICAL-ECONOMIC EVALUATIONS**<br>Dr Kevin ZARCA	**APHP - DRCI - URC Eco Ile de France**<br>http://recherche-innovation.aphp.fr/urc-eco/<br>Hôpital de l'Hôtel Dieu<br>1 Place du parvis Notre Dame, 75004 Paris, France<br>kevin.zarca@aphp.fr<br>Tel : 01.40.27.55.79
Prof Isabelle DURAND-ZALESKI	**Université de Paris, CRESS, INSERM, INRA, URCEco, AP-HP, Hôpital de l'Hôtel Dieu, PARIS**<br>Santé Publique Hôpital Henri Mondor<br>51 avenue du Maréchal de Lattre de Tassigny<br>F 94010 CRETEIL, France<br>Tel 01 40 27 41 43<br>isabelle.durand-zaleski@aphp.fr

TABLE OF CONTENTS

1 ABBREVIATIONS 6

2 SYNOPSIS 7

3 STUDY OVERVIEW 11

4 STUDY FLOWCHART 12

5 SCIENTIFIC RATIONALE OF THE STUDY 13

5.1 STUDY RATIONALE 13
5.2 PROFIT/RISK BALANCE 14
5.2.1 Benefits 14
5.2.2 Risks 14
5.3 EXPECTED IMPACTS 14
5.4 RESEARCH HYPOTHESIS 15

6 STUDY OBJECTIVES 15

6.1 PRIMARY OBJECTIVE 15
6.2 SECONDARY OBJECTIVES 15

7 ENDPOINTS 16

7.1 PRIMARY ENDPOINT 16
7.2 SECONDARY ENDPOINTS 16

8 STUDY DESIGN 17

8.1 METHODOLOGY 17
8.2 STUDY DURATION 17
8.3 SUBJECTS SELECTION 17
8.3.1 Inclusion criteria 17
8.3.2 Non-inclusion criteria 17
8.4 STUDY PLAN 18
8.4.1 Consent sign 18
8.4.2 Randomization procedure 18
8.4.3 Scalp cooling procedures for CIA prevention 18
8.5 SCHEDULE OF ASSESSMENT 19
8.5.1 Pre-randomization inclusion assessment 19
8.5.2 Post-randomization assessment prior to the start of chemotherapy 19
8.5.3 Assessment after each chemotherapy treatment 19
8.5.4 Assessment at the end of the last chemotherapy treatment 19
8.5.5 Follow-up assessment 20
8.6 CONCOMITANT MEDICATION 20

9 STUDY DEVICE 20

9.1 ASSESSMENT OF THE CIA 20
9.2 QUESTIONNAIRES 21
9.2.1 Quality-of-life: EORTC QLQ-C30 and QLQ-BR23 21
9.2.2 Anxiety and depression: HADS 21
9.2.3 Body image: Body Image Scale (BIS) 21
9.2.4 Comfort level of wearing the scalp cooling device 21
9.2.5 Care satisfaction : OUTPATSAT-35 CT 21
9.2.6 Return to work 21

10 SCALP COOLING DEVICE 21

10.1 STANDARD COOLING CAP (CONTROL GROUP) 21
10.1.1 Presentation 21
10.1.2 Setting up procedure 22
10.2 COOLING CAP BY SCALP COOLING: PAXMAN® PSCS 2 (EXPERIMENTAL GROUP) 23
10.2.1 Presentation 23
10.2.2 Setting up procedure 24

11 SAFETY **25**
11.1 DEFINITIONS 25
11.1.1 Adverse Event (AE) *25*
11.1.2 Serious Adverse Events (SAE) *25*
11.2 INVESTIGATIOR RESPONSIBILITIES 26
11.2.1 Methods for detecting and collecting adverse events *26*
11.2.2 Notification of Serious Adverse Events and Medical Device Defects *26*
11.2.3 Special cases *27*
11.3 SPONSOR RESPONSABILITY 28
11.3.1 Reference document for determining expected/unexpected events *28*
11.3.2 Reporting of serious adverse events and device defects by the sponsor to the authorities *28*
11.3.3 New Facts *28*
12 STATISTICAL CONSIDERATIONS **28**
12.1 SAMPLE SIZE 28
12.2 STATISTICAL ANALYSES 29
12.2.1 General considerations *29*
12.2.2 Main objective *29*
12.2.3 Secondary objectives *29*
13 MEDICAL-ECONOMIC EVALUATION **29**
14 TRIAL FOLLOW-UP **30**
15 ETHICS AND REGULATORY CONSIDERATIONS **31**
15.1 CLINICAL TRIAL APPROVALS 31
15.2 INFORMATION OF PATIENTS INVOLVED IN THE RESEARCH 31
15.3 PERSONAL DATA PROTECTION 31
15.4 INVESTIGATOR RESPONSIBILITIES 32
16 DATA AND DOCUMENTS KEEPING **32**
16.1 DATA ENTRY AND HANDLING 32
16.2 ARCHIVING 33
16.3 PUBLICATION POLICY 33
17 FUNDING AND INSURANCE **33**
17.1 FUNDING 33
17.2 INSURANCE 33
18 REFERENCES **33**

1 ABBREVIATIONS

AE	Adverse Event
CFB	Centre François Baclesse
CTCAE	Common Terminology Criteria for Adverse Events
OS	Overall Survival
PFS	Progression-Free Survival
QoL	Quality of Life
RECIST	Response Evaluation Criteria in Solid Tumours
SAE	Serious Adverse Event
SPC	Summary of Product Characteristics

2 SYNOPSIS

**TITLE**	**Prevention of alopecIa in patients with localized breast CancEr using scalp cooLing vs cooling cAp: a raNDomized controlled trial with medical-economic evaluation**
**ACRONYM**	**ICELAND**
**Coordinator**	**Rose-Marie CHARLES**
**INDICATION**	Patient with localized breast cancer treated with neoadjuvant or adjuvant chemotherapy based on anthracyclines and taxanes (3 or 4 Epirubicine-Cyclophosphamide followed by 3 Docetaxel or 9 to 12 Paclitaxel).
**DESIGN**	A prospective, multicenter, randomized, open-label phase III trial comparing the efficacy of automated scalp cooling with that of a standard nonautomated cooling cap in preventing chemotherapy-induced alopecia (CIA) in patients receiving anthracycline- and taxane- based chemotherapy for localized breast cancer
**OBJECTIVES**	❖ **MAIN OBJECTIVE**<br>To compare the efficacy of a scalp-cooling automated device with that of a standard cooling cap (cold-cap) in preventing CIA in patients receiving anthracycline or taxane-based chemotherapy for localized breast cancer<br>❖ **Secondary objectives**<br>To evaluate and compare between the 2 groups:<br>- The degree of CIA in patients before the first course of taxanes by (i) patient self-assessment and (ii) oncologist assessment.<br>- The degree of CIA and hair regrowth in patients with localized breast cancer at the first follow-up consultation by (i) self-assessment and (ii) oncologist assessment.<br>- Patient adherence to the CIA prevention technique<br>- The comfort level of the CIA prevention technique<br>- Body image before the start of chemotherapy treatment, before the first course of taxanes, at the end of chemotherapy, and then during the follow-up consultation<br>- Quality of life and levels of anxiety and depression before the start of chemotherapy treatment, before the first course of taxanes, at the end of chemotherapy, and then during the follow-up consultation<br>- Patient satisfaction with care of at the end of chemotherapy treatment<br>- Return to work and/or daily activities at the time of the first follow-up consultation<br>- The time spent by caregivers setting up, using, and maintaining the cooling device during chemotherapy sessions<br>- The medico-economic impact using cost-utility and cost-effectiveness analysis, with CIA as the effectiveness criterion
**JUDGEMENT CRITERIA**	❖ **MAIN CRITERION**<br>Proportion of patients with CIA of less than 50% of hair mass (corresponding to grade 0-1 according to NCI-CTCAE v.5.0 criteria) between the start and end of the last chemotherapy course (anthracyclines and taxanes), assessed by independent centralized review of two photographs of the hair taken in a standardized manner on the day of the first course (baseline) and at the end of the last chemotherapy course (end of treatment).<br>In summary, the CIA prevention device will be considered **effective** if the patient has lost **less than 50% of her hair mass** (i.e., **grade 0 or 1 alopecia according to CTCAE v5.0 criteria**) at the end of the last chemotherapy course; it will be considered as **ineffective** if the patient has lost **more than 50% of her hair mass** (i.e., **grade 2 alopecia according to CTCAE v5.0 criteria**).

	**❖ Secondary criteria**<br><br>The secondary criteria are:<br>- The proportion of patients with CIA grade 0, 1, 2 (NCI-CTCAE v.5.0 criteria) prior to the first course of taxanes, (i) according to the patient's self-assessment and (ii) the oncologist's assessment.<br>- Grade 0, 1, 2 CIA (NCI-CTCAE v5.0 criteria), duration of CIA, and quality of hair regrowth at the follow-up oncology consultation, as assessed (i) by the patient and (ii) the oncologist.<br>- The proportion of patients discontinuing the cooling device before the end of chemotherapy, the number of courses of treatment with the cooling cap.<br>- The comfort level for various comfort parameters (feeling of cold, headaches, eye or neck pain) after the first use, after the first taxane cycle, and last use of the scalp cooling device.<br>- Body image scores according to the Body Image Scale (BIS) questionnaire administered at inclusion, before the first course of taxanes, at the end of chemotherapy, and during the follow-up consultation.<br>- Quality of life scores according to the EORTC QLQ C30 and QLQ-BR23 self-questionnaires, anxiety/depression scores according to the HADS scale.<br>- Patient satisfaction scores for care according to the Out-PATSAT35 CT self-questionnaire administered at the last course of chemotherapy.<br>- The date and conditions for returning to work and/or daily activities at the follow-up consultation<br>- The time required for setting up, changing cooling caps, removing and disinfecting the devices, assessed by caregivers at each chemotherapy course.<br>- The cost-utility ratio, where utility is defined as quality-adjusted life expectancy (Quality Adjusted Life Years QALY), calculated based on utility measured by the EORTC QLQ C30.<br>- The cost-effectiveness ratio, using as the effectiveness criterion the proportion of patients with CIA grade 0, 1, 2 (according to NCI-CTCAE v5.0 criteria) at the end of the last course of chemotherapy.
**INCLUSION CRITERIA**	- Patient ≥ 18 years old<br>- Female gender<br>- Histologically proven diagnosis of localized, non-metastatic breast cancer<br>- Indication for neo-adjuvant or adjuvant chemotherapy based on anthracyclines and taxanes (3 or 4 EC followed by 3 Docetaxel or 3 or 4 EC plus 9 to 12 Paclitaxel); HER-positive status is not a non-inclusion criterion<br>- Patients agreeing to be photographed for CIA assessment<br>- Proficiency in the French language<br>- Patient affiliated to an appropriate social security system<br>- Patient who has signed informed consent obtained before any trial related activities
**NON INCLUSION CRITERIA**	- History of neck pain<br>- Pre-existing alopecia<br>- Simultaneous participation in another clinical study<br>- Scalp dermatosis<br>- Contraindication to scalp cooling<br>- History of migraines/headaches, stroke, hyperthyroidism<br>- History of scalp metastases<br>- Cold sensibility, cold agglutinin disease, cryoglobulinemia, cryofibrinogenemia or post-traumatic cold dystrophy<br>- Malignant blood disorders<br>- History of chemotherapy treatment

	- Indication for brain irradiation<br>- Raynaud's syndrome<br>- Pregnant or breastfeeding woman<br>- Patient assessed by the investigator to be unable or unwilling to comply with the requirements of the protocol<br>- Patient deprived of liberty or placed under the authority of a tutor
**EXPERIMENTAL PLAN**	✓ **Information, inclusion, randomization:**<br>This study will be proposed to eligible patients by nurses during the pre-chemotherapy interview or by oncologists during the medical consultation prior to chemotherapy. The duration of chemotherapy is 4 to 5 months (3 to 4 courses of EC, followed by 3 courses of docetaxel or 9 to 12 courses of paclitaxel).<br>After obtaining their consent, patients will be assigned, according to a 1:1 randomization, stratified by chemotherapy regimen, center and menopausal status, to either:<br>✓ Standard arm: standard cooling cap (cold-cap)<br>✓ Experimental arm: scalp-cooling automated technique<br><br>✓ **Intervention:**<br>After randomization, the scalp cooling device will be administered during each chemotherapy treatment, according to the specific characteristics of each cap and chemotherapy regimen.<br><br>✓ **Standard arm:** standard cooling cap (cold-cap)<br>Patients will benefit from prevention of CIA through the use of a standard cooling cap device (cold-cap) throughout their anthracycline- and taxane-based chemotherapy treatment.<br>This device will be supplemented by a compression bandage to enhance the vasoconstrictive effect of the cold and the compression of the scalp by the bandage, thereby optimizing device’s effectiveness.<br><br>✓ **Experimental arm:** scalp-cooling technique<br>Patients will benefit from prevention of CIA through the scalp-cooling device throughout chemotherapy treatment based on anthracyclines and taxanes.<br><br>For each of the two groups, cooling devices will be:<br>✓ Applied at least 15 minutes before the start of the infusion (up to 30 minutes depending on the chemotherapy regimen and device),<br>✓ Kept in place throughout the infusion (depending on the injection duration, one or more changes of cold-cap (standard arm) will be necessary),<br>✓ Left in place for at least 20 minutes after the end of the infusion (up to 120 minutes depending on the chemotherapy regimen and device)<br>✓ In consultation with the doctor, 1 g of paracetamol may be prescribed for each course of treatment to prevent the risk of cooling device-induced headaches.<br><br>✓ **Assessments:**<br>The assessments will be conducted identically in both groups.<br>✓ **Pre-randomization inclusion assessment:**<br>o Quality of life assessment: EORTC QLQ-C30 questionnaire and its additional BR23 module<br>o Anxiety/depression assessment: HADS scale<br>o Body image assessment: Body Image Scale (BIS)<br><br>✓ **Post-randomization assessment before the start of chemotherapy:**<br>o Standardized photograph of the scalp (photograph 1)<br><br>✓ **During chemotherapy:**<br>✓ *At each course:*<br>o Visual assessment of hair by the nurse or oncologist (in accordance to standard practice)

<table>
<tr><td></td><td>

In the event of refusal to wear the cooling cap for treatment (course ≥ 2), the nurse shall record the reasons given by the patient
At the end of infusion:

Assessment by the patient of the cooling device’s comfort level (questionnaire), for the 1st course only.
Recording by the caregiver of the time taken to set up, replace cooling caps, remove and disinfect the devices.

✓ <u>Before the first course of taxanes:</u>

Visual self-assessment of the CIA by the patient, and assessment by the nurse or oncologist.
Quality of life assessment: EORTC QLQ-C30 questionnaire and its additional BR23 module
Anxiety/depression assessment: HADS scale
Body image assessment: BIS (Body Image Scale)

✓ <u>At the last chemotherapy course:</u>

Quality of life assessment: EORTC QLQ-C30 questionnaire and its additional BR23 module
Anxiety/depression assessment: HADS scale
Body image assessment: BIS (Body Image Scale)
Taking a standardized photograph of the scalp (photograph 2)
Assessment of patient satisfaction with care: Out-PATSAT35 chemotherapy self-questionnaire
Patient assessment of the comfort level of the cooling device (questionnaire)

✓ Monitoring after chemotherapy:
Follow-up assessment 6 months after the end of chemotherapy (roughly corresponding to the first oncological follow-up consultation) will include:

Visual assessment by the oncologist and the patient, of the CIA grade and the quality of hair regrowth.
Quality of life assessment: EORTC QLQ-C30 questionnaire and its additional BR23 module
Anxiety/depression assessment: HADS scale
Body image assessment: BIS (Body Image Scale)
Assessment of return to work and/or daily activities (questionnaire)
Assessment of coverage for the cost of hair prostheses and outpatient consultations related to alopecia

</td></tr>
<tr><td>SAMPLE SIZE</td><td>196 patients (98 per arm)</td></tr>
<tr><td>PARTICIPATING CENTERS</td><td>3 French comprehensive cancer centers :

François Baclesse Center (CAEN)
Oscar Lambret Center (LILLE)
IUCT-Oncopole (TOULOUSE)
</td></tr>
<tr><td>STUDY DURATION</td><td>Estimated recruitment period: 48 months
Duration of each patient’s participation: 12 months
Duration of study: 60 months
Start date of research: 09/08/2023
End date of research: 09/08/2028</td></tr>
</table>

3 STUDY OVERVIEW

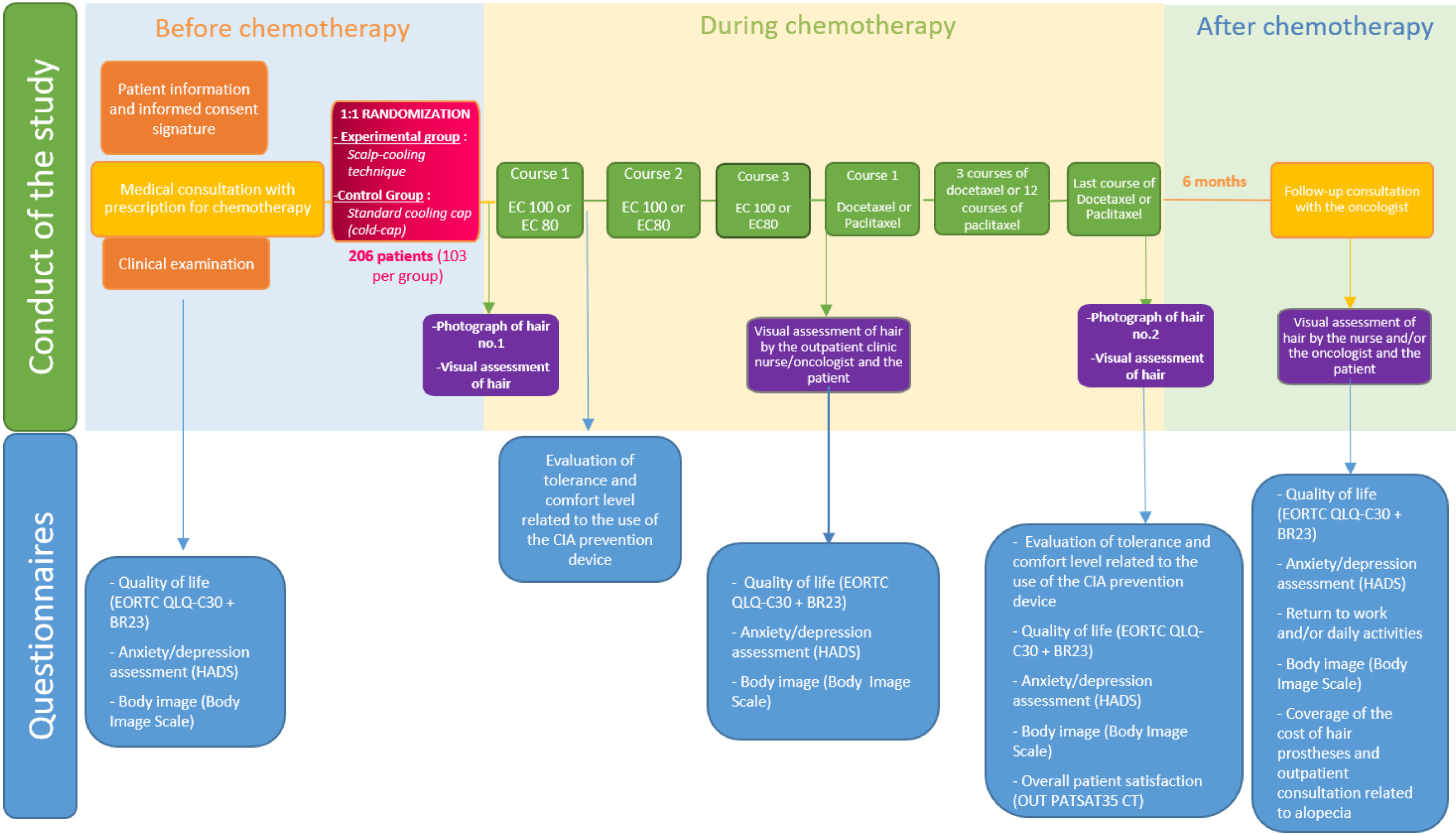

4 STUDY FLOWCHART

	INCLUSION	COURSE 1 ANTHRACYCLINES EC	COURSE 1 TAXANES *(Docetaxel or Paclitaxel)*	LAST CURE TAXANES *(Docetaxel or Paclitaxel)*	FOLLOW-UP CONSULTATION (at 6 months)
**Patient information and informed consent signature**	•				
**Clinical examination** (history of illness, medical history, weight, height, vital signs)	•				
**Quality of life assessment** (EORTC QLQ-C30 and QLQ BR23 self-questionnaires)	•		•	•	•
**Anxiety and depression assessment** (HADS scale)	•		•	•	•
**Body image assessment** (Body Image Scale, BIS)	•		•	•	•
**Assessment of the tolerance and the comfort level of the device**		•	•	•	
**Hair photograph**		•		•	
**Hair visual assessment by the patient and the oncologist**			•	•	•
**Return to work and/or daily activities** (questionnaire)					•
**Overall patient satisfaction** (OUT-PATSAT35 CT self-questionnaire)				•	
**Evaluation of the coverage of the cost of hair prostheses and outpatient consultations related to alopecia**					•

5 SCIENTIFIC RATIONALE OF THE STUDY

5.1 STUDY RATIONALE

Localized breast cancer affects more than 58,000 women in France every year. In approximately 25% of cases, standard treatment includes anthracycline- and taxane-based chemotherapy. The duration of chemotherapy is 4 to 5 months (3 to 4 courses of EC, followed by 3 courses of Docetaxel or 9 to 12 courses of Paclitaxel). These treatments cause significant hair loss and are known to lead to chemotherapy-induced alopecia (CIA) in most patients, in the absence of any preventive devices.

CIA is mostly temporary, but can persist in 10% of cases [Martin, 2018]. Hair regrowth usually begins 4 to 6 weeks after the last chemotherapy treatment but may be slower or suboptimal in cases of treatment with anthracyclines or taxanes. For patients and their loved ones, hair loss is often seen as a symbol of cancer and sometimes the only outward sign of the disease. Therefore, CIA is one of the least tolerated side effects of chemotherapy. It can have significant psychosocial consequences, with a negative impact on self-image and psychosocial well-being [Lemieux, 2008]. Prevention of CIA remains one of the major levers for improving the tolerance and acceptability of cytotoxic treatments in these patients.

Scalp cooling and compression devices are the only techniques with proven effectiveness in reducing and preventing CIA [Zhou T, 2021]. They are suitable for chemotherapy administered by intravenous infusion in the treatment of solid tumors. They are also contraindicated for hematologic cancers, in cases of scalp metastases, and in patients with brain tumors. The safety assessment of cooling techniques showed no increased risk of scalp metastases [Betticher, 2013].

These cooling techniques are based on the compressive application of cap-type helmets over the entire scalp. The compressive application of these caps reduces blood flow to the scalp. Cooling causes vasoconstriction, contributing to a reduction in the quantity of cytotoxic agents in the hair follicles, and thus to a lower risk of damage to these follicles. It can also decrease the mitotic activity of hair follicles, thereby helping to make them less sensitive to the toxic effects of chemotherapy.

Beyond its preventive effect on CIA, cooling preserves hair follicles from the effects of chemotherapy, improves the precocity and homogeneity of hair regrowth. This beneficial effect of cooling on hair regrowth is even more important to consider when administering taxanes, which can lead to poorer quality regrowth.

Two types of caps are mainly used for scalp cooling.

The standard nonautomated cooling cap (*cold cap*) is a glycerine gel cap (stored at a minimum temperature of -20°C) applied directly to the scalp. The application, combined with an intense cold sensation (around -15°C), must be repeated every 30 to 45 minutes to maintain the device's effectiveness.

The automated *scalp cooling* technique involves the use of caps (from Paxman® society) connected to a refrigeration unit that circulates a cooling liquid inside the cap. It allows continuous cooling of the scalp at a temperature of -4°C, limiting thermal chock.

The use of cooling caps may remain limited in certain establishments due to a lack of conviction among caregivers. Cooling caps have suffered from a poor reputation regarding their effectiveness and patient tolerance (headaches, feeling of extreme cold, etc.). In the collective belief, these caps do not always prevent chemotherapy-induced alopecia but, in the best cases, simply reduce or delay it. Overall, this may contribute to reinforcing the negative view of the device, namely an increase in workload without convincing results.

In current clinical practice, there are still barriers to the use of cooling caps. From an organizational standpoint, implementing cooling caps requires nursing time and logistics. The use of cooling caps requires additional nursing time, which is difficult to find in the current context of a shortage of healthcare professionals, resulting in a prioritization of medical care and the relegation of supportive care to the background. From a logistical standpoint, the use of cooling caps also requires storage in a room equipped for cleaning and drying, as well as a freezer for glycerin gels. Scalp-cooling devices require more time to apply before and after the administration of intravenous chemotherapy. It therefore seems important to measure the costs associated with ACI prevention devices. Measuring the medical and economic impact of each of these devices is necessary to inform the choices made by professionals and their institutions.

5.2 PROFIT/RISK BALANCE

5.2.1 Benefits

Most patients who are candidates for chemotherapy for localized breast cancer are apprehensive about chemotherapy-induced alopecia (CIA), which is associated with an outward sign of the disease. The present study proposes that all patients, regardless of their randomization group, wear a scalp cooling device to prevent CIA.

The expected benefits are therefore similar for all patients. Wearing a cooling cap should better prevent the onset of CIA, improve their quality of life and self-image, and enable them to better accept chemotherapy treatment, thereby contributing to a lesser impact of the disease and its treatments on their social life (daily and /or professional activities).

Furthermore, the cost of hair protheses very often exceeds the amount covered by health insurance: reducing the degree of CIA could effectively reduce the use of hair prostheses, thereby reducing the amount patients have to pay out of pocket. CIA is one of the obstacles to returning to work: better prevention of ACI and earlier hair regrowth could therefore help to encourage a return to work and/or daily activities.

5.2.2 Risks

Wearing a cap can cause some discomfort and inconvenience: the sensation of cold (-15°C for standard cold caps; -4°C for scalp-cooling caps) can, in some cases, lead to migraines, eye pain, and/or neck pain.

In addition, the conditions for using scalp cooling require wearing the cap for nearly 3 hours per chemotherapy session, covering the duration of the infusion, for optimal effectiveness. In addition, scalp cooling must be worn for nearly 3 hours per chemotherapy session, covering the duration of the infusion, for optimal effectiveness. For standard cooling caps, the cap must be replaced every 45 minutes to ensure scalp cooling.

5.3 EXPECTED IMPACTS

Prevention of CIA is still not adequately addressed in France. Strengthening evidence on the benefits of CIA prevention strategies, based on robust methodologies, remains a necessary prerequisite for better integration of appropriate supportive care for patients receiving chemotherapy.

The expertise and the extensive experience of the Oscar Lambret Center in the prevention of CIA through the use of cooling caps (cold cap) has been shared with the François Baclesse Center. These exchanges have enabled a proactive approach to CIA prevention: the genesis and reflection around the project proposed here have already enabled healthcare teams to better take this side effect into account and improve care.

This research should provide new insights into the benefits of scalp cooling during anthracycline- and taxane-based chemotherapy for preventing CIA, for each of the two cooling techniques. In addition to its effectiveness in preventing CIA, quality of life, self-image, and satisfaction with care will be assessed by patients during and after chemotherapy. The medico-economic aspects will also be evaluated for each of the two cooling modalities. The results of the various proposed evaluations will help guide the choice between these two CIA prevention techniques.

5.4 RESEARCH HYPOTHESIS

We found few randomized studies on the impact of scalp cooling in preventing CIA, in which the control group did not use any scalp cooling technique. In particular, we did not find any randomized controlled studies comparing scalp cooling with standard cooling caps. Furthermore, few of these studies included patients receiving anthracycline-based chemotherapy [Betticher, 2013; Rugo, 2017; Rugo, 2018; Zhou T, 2021].
Scalp cooling technology has been around for 20 years. It has been approved by the Food Drug Administration (FDA) since 2015. In 2019, it was added as a category 2A recommendation for the prevention of CIA in breast cancer in the NCCN guidelines (National Comprehensive Cancer Network Clinical Practice Guidelines in Oncology, version 1, 2019). However, most French centers currently limit themselves to using standard cooling caps.
We therefore wish to propose a randomized study that will compare the effectiveness of these two scalp cooling techniques in preventing alopecia in patients receiving anthracycline- and taxane-based chemotherapy for localized breast cancer. A medical-economic evaluation of each of the two prevention methods is also planned.

6 STUDY OBJECTIVES

6.1 PRIMARY OBJECTIVE

The primary objective is to compare the effectiveness of a scalp-cooling device with that of a standard cooling cap (cold cap) in preventing CIA in patients receiving anthracycline- and taxane-based chemotherapy for localized breast cancer.

6.2 SECONDARY OBJECTIVES

The secondary objectives are to evaluate and compare between the 2 groups:

- The degree of CIA in patients before the first course of taxanes by (i) patient self-assessment and (ii) oncologist assessment.
- The degree of CIA and hair regrowth in patients with localized breast cancer at the first follow-up visit by (i) self-assessment and (ii) oncologist assessment.
- Patient adherence to the ACI prevention technique
- Level of comfort with the ACI prevention technique
- Body image before the start of chemotherapy treatment, before the first course of taxanes, at the end of chemotherapy, and then during the follow-up consultation
- Quality of life and levels of anxiety and depression before the start of chemotherapy treatment, before the first course of taxanes, at the end of chemotherapy, and then during the follow-up consultation
- Patient satisfaction with care at the end of chemotherapy treatment
- Return to work and/or daily activities at the time of the first follow-up consultation

- Time spent by caregivers setting up, using, and maintaining the cooling device during chemotherapy sessions
- Medical and economic impact using cost-utility and cost-effectiveness analyses, with CIA as the effectiveness criterion

7 ENDPOINTS

7.1 PRIMARY ENDPOINT

The primary endpoint of the study is the proportion of patients with less than 50% hair loss of the hair mass (corresponding to grade 0-1 according to NCI-CTCAE v.5.0 criteria) between the start and end of the last chemotherapy treatment (anthracyclines and taxanes), assessed by independent centralized review (see section 9.1) of two photographs of the hair taken in a standardized manner on the day of the first course (baseline) and at the end of the last course of chemotherapy (end of treatment).

In summary, the CIA prevention regimen will be considered effective if the patient experiences less than 50% hair loss (i.e., grade 0 or 1 alopecia according to CTCAE v5.0 criteria) at the end of the last chemotherapy treatment; it will be considered ineffective if the patient experiences a loss of more than 50% of her hair mass (i.e., grade 2 alopecia according to CTCAE v5.0 criteria).

7.2 SECONDARY ENDPOINTS

The secondary endpoints are the following:

- The proportion of patients with grade 0, 1, or 2 CIA (NCI-CTCAE v.5.0 criteria) before the first course of taxanes, (i) according to the patient's self-assessment and (ii) according to the oncologist's assessment.
- The grade of CIA (according to NCI-CTCAE v.5.0 criteria: grade 0 [no hair loss], grade 1 [hair loss <50% compared to inclusion] or grade 2 [hair loss ≥50% compared to inclusion]), the duration of CIA and the quality of hair regrowth at the oncology follow-up consultation assessed (i) by the patient and (ii) by the oncologist.
- The proportion of patients abandoning the cooling device before the end of chemotherapy, the number of treatments with the cooling cap.
- The level of comfort through a 4-point Likert scale for various comfort parameters (comfort with regards to the scalp cooling device, cold sensation, and duration of scalp cooling) after the first use, after the first taxane cycle, and last use of the scalp cooling device.
- Body image scores according to the Body Image Scale (BIS) questionnaire administered at inclusion, before the first taxane treatment, at the end of chemotherapy, and during the follow-up consultation.
- Quality of life scores according to the EORTC QLQ C30 and QLQ-BR23 self-administered questionnaires, anxiety/depression scores according to the HADS scale
- Patient satisfaction scores based on the OUT-PATSAT35 CT self-administered questionnaire administered at the last chemotherapy treatment
- Date and conditions of return to work and/or daily activities at the follow-up consultation.
- The time required to set up, change cooling caps, remove, and disinfect devices, as assessed by caregivers during each chemotherapy treatment
- The cost-utility ratio, with utility being quality-adjusted life years (QALY) calculated based on utility measured by the EORTC QLQ C30

- The cost-effectiveness ratio, using as the effectiveness criterion the proportion of patients with a grade 0-1 CIA (according to NCI-CTCAE v.5.0 criteria) at the end of the last chemotherapy treatment

8 STUDY DESIGN

8.1 METHODOLOGY

We propose a prospective, multicenter, randomized, open-label phase III trial comparing the efficacy of scalp cooling with that of standard cooling caps in preventing chemotherapy-induced alopecia (CIA) in patients receiving anthracycline- and taxane-based chemotherapy for localized breast cancer.

8.2 STUDY DURATION

The estimated duration of recruitment is 48 months in three cancer centers.

The duration of the intervention for the prevention of CIA by scalp cooling corresponds to the duration of chemotherapy (between 4 and 5 months): 3 or 4 courses of EC, followed by 3 courses of docetaxel or 9 to 12 courses of paclitaxel.

Patients will be followed up for up to 6 months after their last chemotherapy treatment, which corresponds approximately to the first follow-up consultation.

The duration of each patient's participation is therefore approximately 12 months.

In total, the estimated duration of the study is 60 months. The start date of the study is estimated as 08/09/2023 and the end date as 08/09/2028.

8.3 SUBJECTS SELECTION

8.3.1 Inclusion criteria

- Age ≥ 18 years old
- Female sex
- Histologically proven diagnosis of localized non-metastatic breast cancer
- Indication for neoadjuvant or adjuvant chemotherapy based on anthracyclines and taxanes (3 or 4 courses of EC, followed by 3 courses of docetaxel or 9 to 12 courses of paclitaxel); HER-positive status is not a non-inclusion criterion
- Patient agrees to be photographed to assess CIA
- Proficiency in French
- Patient affiliated to an appropriate social security system
- Signature of informed consent

8.3.2 Non-inclusion criteria

- History of neck pain
- Pre-existing alopecia
- Participation in another trial
- Scalp dermatosis
- Contraindication to scalp cooling
- History of migraines/headaches, stroke, hyperthyroidism
- History of scalp metastases
- Cold sensitivity, cold agglutinin disease, cryoglobulinemia, cryofibrinogenemia or post-traumatic cold dystrophy
- Malignant blood disorders
- History of chemotherapy treatment
- Indication for brain irradiation

- Pregnant or breastfeeding patient
- Raynaud's syndrome

8.4 STUDY PLAN

8.4.1 Consent sign

The study is proposed to patients meeting the eligibility criteria, by nurses during the pre-chemotherapy interview or by oncologists during the medical consultation prior to chemotherapy. Patients are given study information (oral explanation and written information notice). Patients have a reflection period of the duration of their choice. The consent form must then be signed by the patient and physician.

After collection of the signed informed consent, the selection criteria are verified before inclusion in the trial.

The specific examinations required for the study prior to inclusion (inclusion assessment) will be performed after signing the consent form and before inclusion.

8.4.2 Randomization procedure

After collection of the signed informed consent, checking of all eligibility criteria and pre-randomization assessment, randomization is performed on the software dedicated to the study via an internet portal. A randomization number is assigned to the patient to be used during the whole study.

Randomization is performed before the initiation of the study intervention, using the ad hoc module of the eCRF / Ennov Clinical® software.

Randomization will be performed in a 1:1 ratio using a centralized computerized procedure based on minimization technique, stratified according to chemotherapy regimen, center and menopausal status.

The patients will be randomized into one of the 2 following arms (ratio 1:1):

- **Control group**: standard cooling cap (cold cap)

  or

- **Experimental group**: scalp-cooling technique

8.4.3 Scalp cooling procedures for CIA prevention

After randomization, the scalp cooling device will be administered during each chemotherapy cure, according to the specific characteristics of each cap and chemotherapy regimen. For each two groups, cooling procedures will be:

- Applied at least 15 minutes before the start of the infusion (up to 30 minutes depending on the chemotherapy regimen and device),
- Kept in place for the entire duration of the infusion (according to the injection duration, one or more changes of cold-cap (standard arm) will be necessary)
- Left in place for at least 20 minutes after the end of the infusion (up to 120 minutes depending on the chemotherapy regimen and device).

Details of the intervention for preventing CIA are specified below for each of the two groups in the section "Scalp cooling devices".

With the doctor's approval, 1g of paracetamol may be prescribed for each treatment to prevent the risk of migraines caused by the cooling devices.

8.5 SCHEDULE OF ASSESSMENT

A summary table of the investigations requested is provided at the beginning of the protocol.

8.5.1 Pre-randomization inclusion assessment

The baseline assessment will be performed prior to randomization and will include:

- A quality-of-life assessment: EORTC QLQ-C30 questionnaire and its additional BR23 module
- An anxiety/depression assessment using the HADS scale
- A body image assessment using the Body Image Scale (BIS)

8.5.2 Post-randomization assessment prior to the start of chemotherapy

Before the first chemotherapy course, the following will be performed:

- A standardized scalp photograph (photograph 1)

8.5.3 Assessment after each chemotherapy treatment

<u>At each cycle of chemotherapy</u>, the following evaluations are realized in day hospital:

- ✓ A visual scalp assessment by the nurse (in accordance with current practice)
- ✓ If the patient refuses to wear the cooling cap for treatment, the nurse shall record the reasons given by the patient.
- ✓ At the end of infusion:
  - Patient assessment of the comfort level of the cooling device, for the 1st course and first course of taxanes, only
  - Recording by caregivers of the time taken to put on, change (if necessary), remove, and disinfect cooling caps.

<u>Before the first course of taxanes</u>, the assessment will also include:

- ✓ A visual self-assessment of the CIA by the patient, and assessment by the nurse and/or oncologist
- ✓ A quality-of-life assessment: EORTC QLQ-C30 questionnaire and its additional BR23 module
- ✓ An anxiety/depression assessment using the HADS scale
- ✓ A body image assessment using the Body Image Scale (BIS)

8.5.4 Assessment at the end of the last chemotherapy treatment

During the last administration of chemotherapy, the following evaluations are realized:

- ✓ A quality-of-life assessment: EORTC QLQ-C30 questionnaire and its additional BR23 module
- ✓ An anxiety/depression assessment using the HADS scale
- ✓ A body image assessment using the Body Image Scale (BIS)
- ✓ A standardized scalp photograph (photograph 2)
- ✓ An assessment of patient satisfaction with care based on the OutPATSAT35 chemotherapy self-assessment questionnaire
- ✓ Patient assessment of the comfort level of the cooling device (using the same methodology as for the first treatment)

8.5.5 Follow-up assessment

A follow-up assessment will be carried out 6 months after the end of chemotherapy, approximately at the time of the first oncology follow-up consultation. It will include:

- A visual assessment, by the oncologist, an APN (Advanced Practice Nurse) or a nurse, and by the patient, of the grade of the CIA and the quality of hair regrowth.
- A quality-of-life assessment: EORTC QLQ-C30 questionnaire and its additional BR23 module
- An anxiety/depression assessment using the HADS scale
- A body image assessment using the Body Image Scale (BIS)
- An assessment of the cost coverage for hair prostheses and outpatient consultations related to alopecia (self-administered questionnaire)

8.6 CONCOMITANT MEDICATION

This study does not alter the therapeutic management of the patients, in any way. The chemotherapy regimens administered will be recorded (including any dose adjustments or modifications).

9 STUDY DEVICE

9.1 ASSESSMENT OF THE CIA

To meet the main objective, CIA will be assessed using two photographs, one taken prior to the start of the first treatment (baseline) and the other at the end of the last treatment (end of therapy). The photographs will be taken in a standardized manner, i.e., as follows:

- Hair will be loosened beforehand if necessary
- The image will show the top of the head
- The patient is seated, the caregiver is standing, and the photograph is taken from a distance of approximately 20 cm.
- The photograph does not show the face at all.

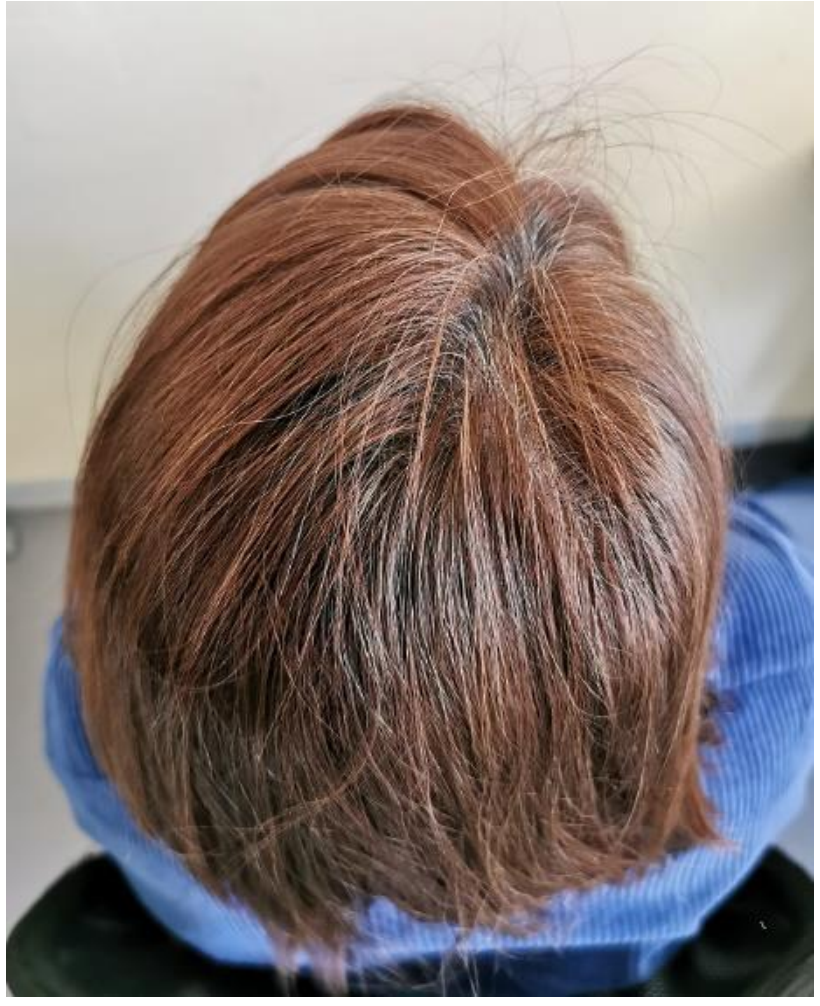

The degree of CIA (preservation of more than 50% of hair, i.e. alopecia ≤ 1 according to CTCAE v5.0 criteria) will be evaluated by an independent committee comprising a medical oncologist, a nurse, and a socio-aesthetician, by comparing the two photographs.
To meet the secondary objectives, the degree of CIA will be measured by a self-assessment by the patient and by a visual assessment by the oncologist.

9.2 QUESTIONNAIRES

9.2.1 Quality-of-life: EORTC QLQ-C30 and QLQ-BR23

The quality of life of the patients will be measured using the EORTC QLQ-C30 questionnaire and its additional QLQ-BR23 module specific to breast cancer. The QLQ-C30 questionnaire contains 30 items exploring 5 functional subscales, 9 symptom subscales and 1 overall quality of life subscale. The QLQ-BR23 module contains 23 items divided into 4 functional subscales (exploring body image, sexual activity, sexual pleasure and future prospects) and 4 symptomatic subscales (exploring side effects, breast symptoms, arm symptoms and concerns about hair loss). The response system uses a Likert-type scale, allowing overall scores to be obtained by summing the items, or by subscales.

9.2.2 Anxiety and depression: HADS

The Hospital Anxiety and Depression Scale (HADS) is a self-questionnaire used to screen for anxiety and depressive disorders. It consists of 14 items, 7 of which relate to anxiety and 7 to depression.

9.2.3 Body image: Body Image Scale (BIS)

The body image representation will be measured by the « Body Image Scale » (BIS) questionnaire. This questionnaire of 10 items, created by Hopwood *et al*. [2001], answers the patient on its perception of its physical appearance and the changes inducted by cancer and treatments. This questionnaire has been validated in French language [Brédart, 2007]. The total score ranges from 0 to 30 after adding up the responses to the 10 items (scored from 0 “Not at all” to 3 “Very often”).

9.2.4 Comfort level of wearing the scalp cooling device

The comfort level of wearing the cooling device will be assessed through a 4-point Likert scale on several parameters (comfort with regards to the scalp cooling device, cold sensation, and duration of scalp cooling).

9.2.5 Care satisfaction : OUTPATSAT-35 CT

During this study, patient satisfaction with the care received will be assessed using the OUTPASAT 35 questionnaire. This questionnaire consists of 35 items with a five-point Likert scale response system (1: poor, to 5: excellent). The OUTPATSAT 35 questionnaire consists of 12 subscales: two sections (doctors and nurses) each comprise 4 subscales (technical skills, quality of information provided, interpersonal skills, and availability). A third section (care organization) includes the following subscales: communication quality between professionals, quality of information provided by other staff, waiting times and accessibility/ physical environment.

9.2.6 Return to work

Patients will be asked about their possible return to work, in terms of the date of return and the conditions of return (full-time or part-time, same position as before the work stoppage, adaptation of the position, completion or scheduling of a dedicated “Occupational Health” consultation, etc.).

10 SCALP COOLING DEVICE

10.1 STANDARD COOLING CAP (CONTROL GROUP)

10.1.1 Presentation

The cooling cap used for patients assigned to the control group will be the cap shown in the photo below.

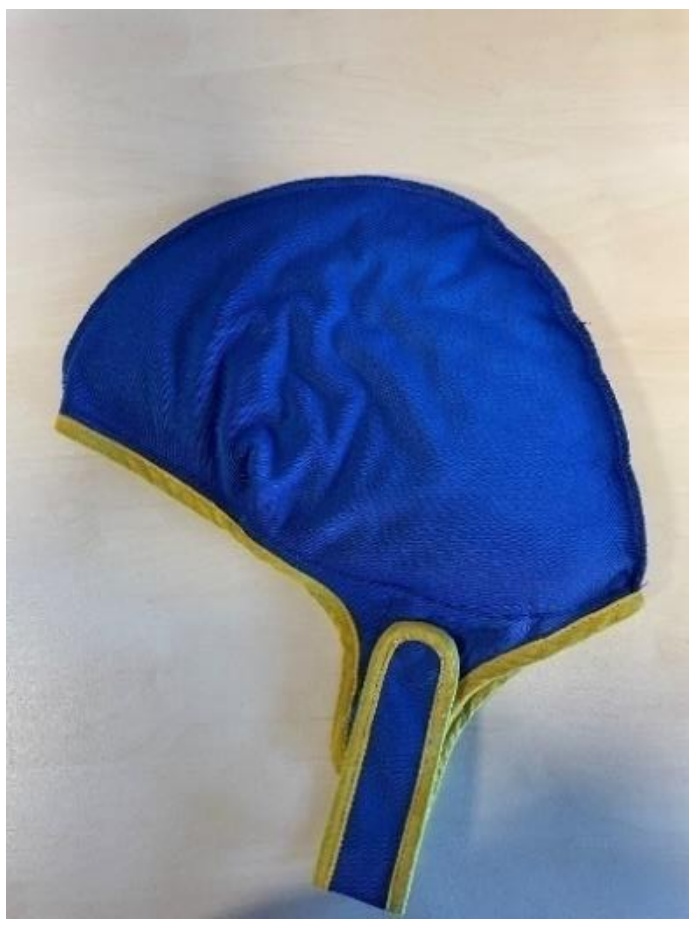

The device consists of a cap with a pocket filled with glycerin gel. Its cooling effect is achieved by placing the pocket in the freezer for at least 12 hours at a temperature of at least -20°C.
In accordance with the standardized care practices of participating centers, and to optimize the use of the standard cap, the use of this device will be supplemented by a compression bandage in order to add the compressive effect of the bandage to the vasoconstrictive effect of the cold.
The duration of wear for the cap supplemented by the compression bandage will comply with the manufacturer's recommendations, depending on the molecules used, as described in the following table:

Treatment	Minimal pre-cooling time	Infusion time	Minimal post-cooling time
EC	**15 minutes +**	Time required for drug infusion	**+ 20 minutes**
Daily Paclitaxel	**15 minutes +**	Time required for drug infusion	**+ 20 minutes**
Docetaxel	**15 minutes +**	Time required for drug infusion	**+ 20 minutes**

The cap would be changed (and the compression bandage replaced) every 45 minutes, as quickly as possible to avoid a vasodilation reflex in the scalp. This information is taken from the manufacturer's instructions of the cooling cap used in the 3 centers.

10.1.2 Setting up procedure

The cold-cap technique will be used according to the following principles, and as illustrated below:

- Choose a cap that fits the patient properly and wait until the last moment to take it out of the freezer
  Caution: The cap effectiveness depends on choosing the right size (risk of uneven alopecia)
- Lightly dampen the cap with cold water, wring it out, and place it on the patient's scalp.
- Place the cotton wool on the forehead and ears to protect them from the cold.
- Leave the cap in place for at least 15 minutes before the infusion and at least 20 minutes after the end of the infusion, according to the recommendations detailed above.
  Example: For an EC, start cooling 15 minutes before Epirubicin and finish 20 minutes after the end of Cyclophosphamide.
- Materials needed:
  - 2 strips of crepe paper measuring 5m x 20cm and 5m x 15cm,
  - 1 woven hairnet,
  - a cooling cap,
  - cotton wool.
- Place the bandage over the cap. The straps are tightened with the patient's assistance.
  A training video on how to put on the cooling caps will be available to nurses.

The device (cap and compression bandage) will be changed every 45 minutes.

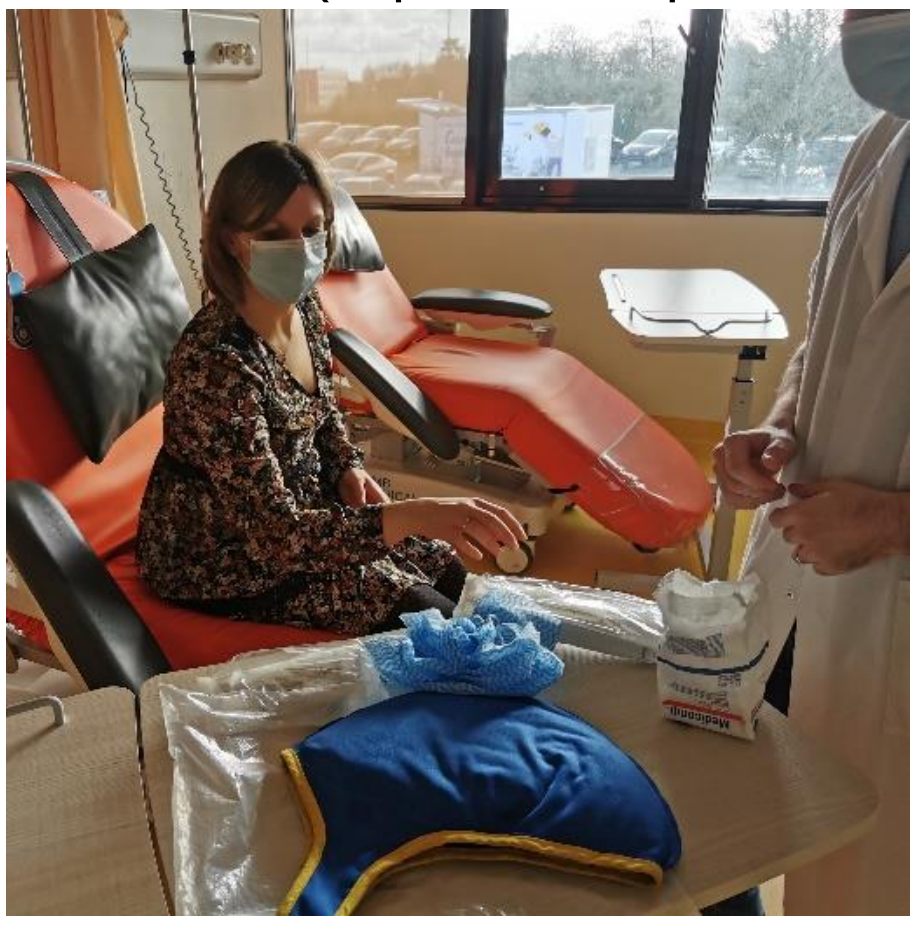
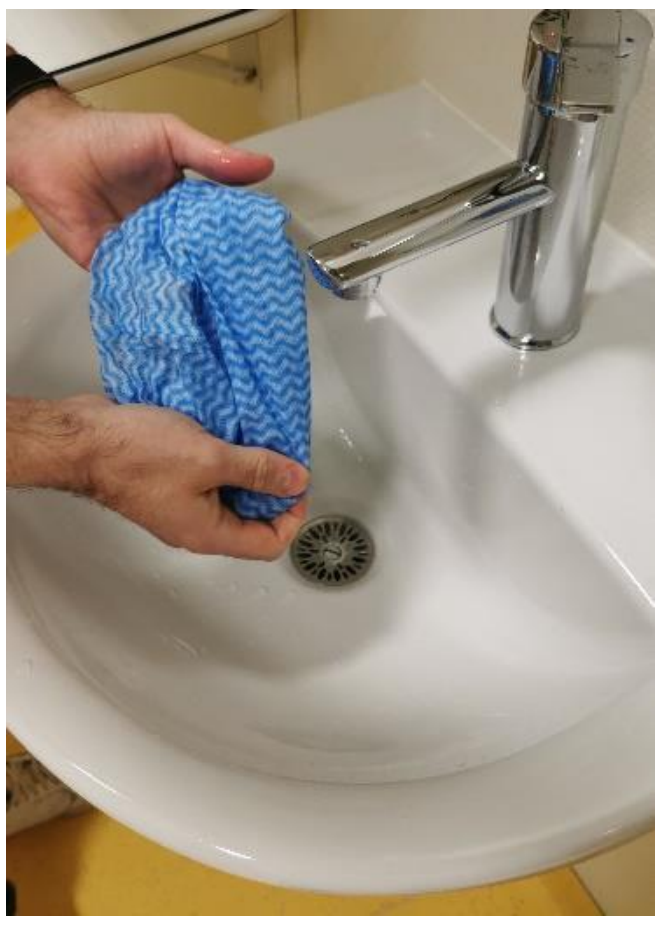
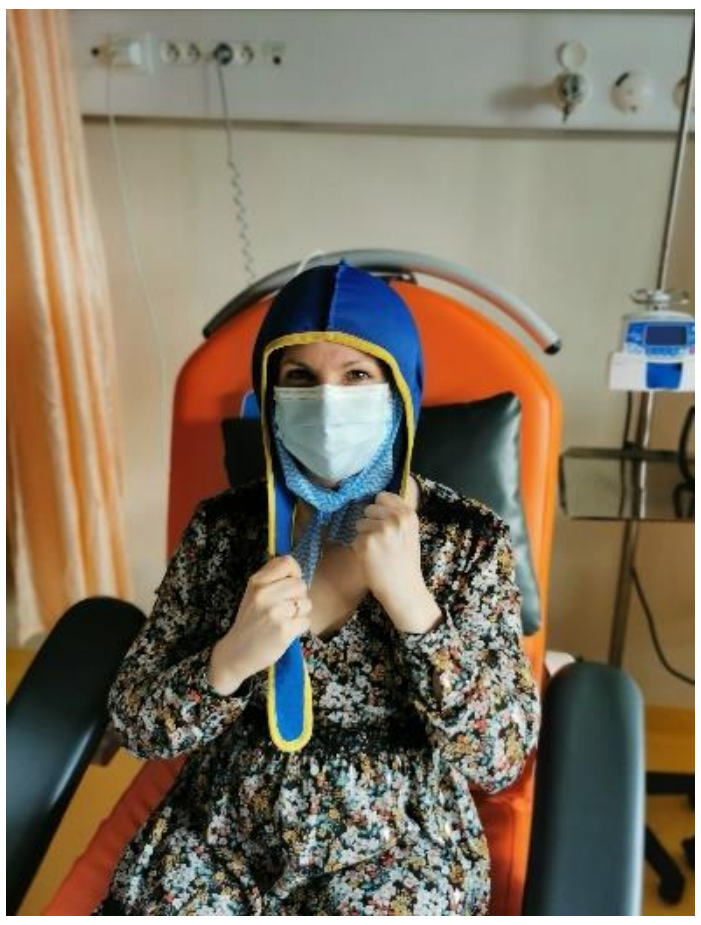
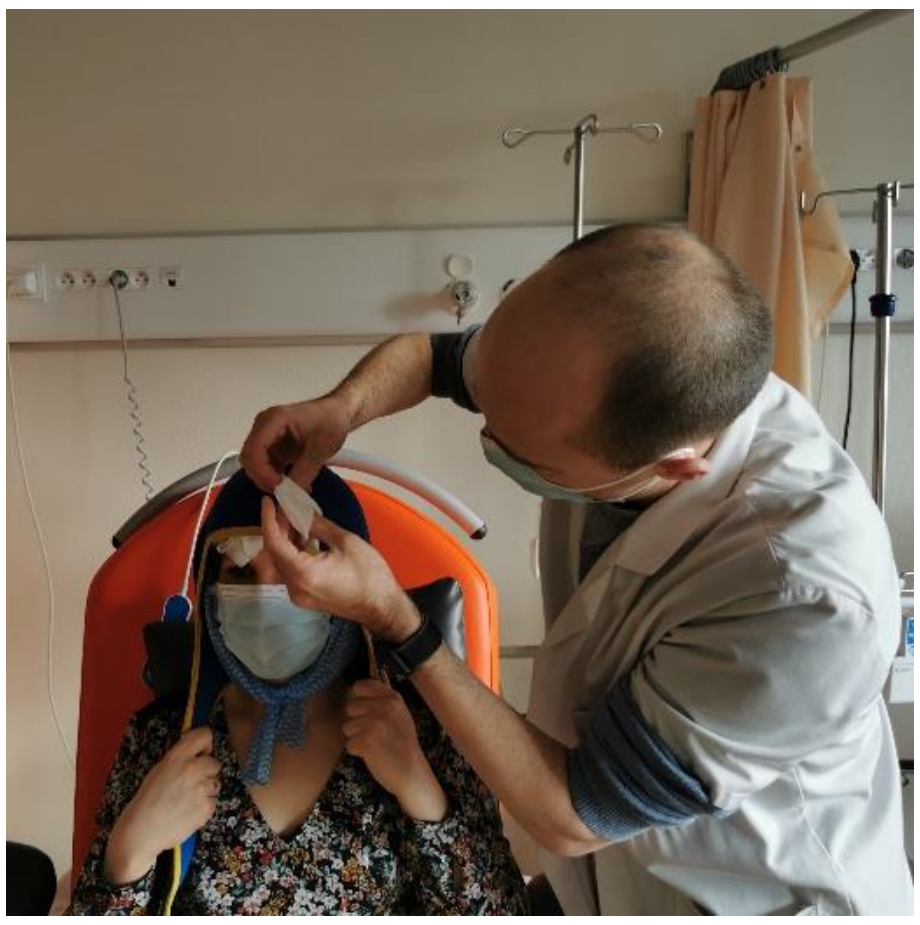
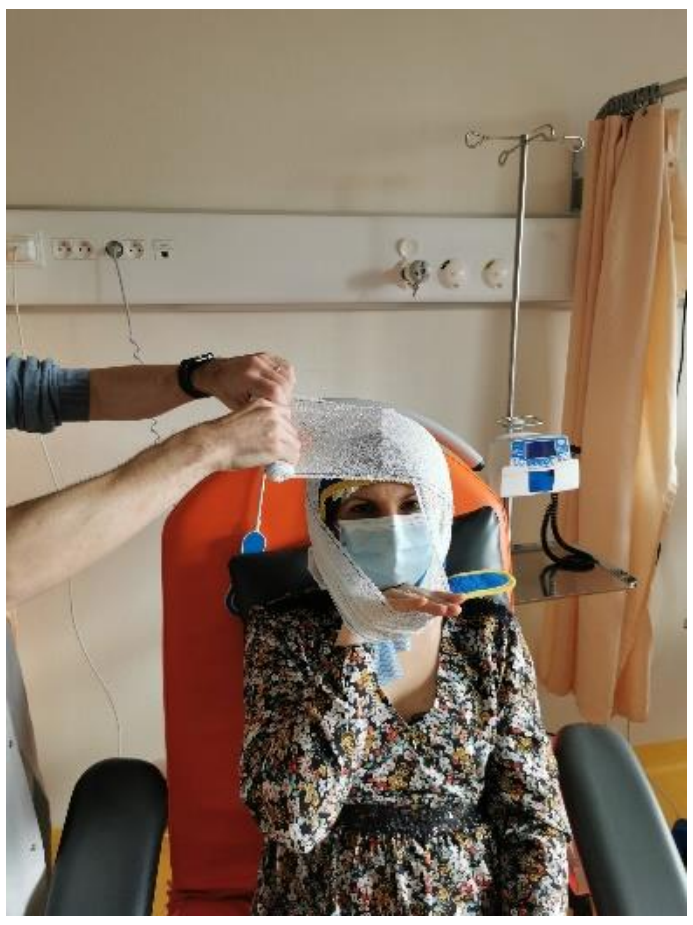
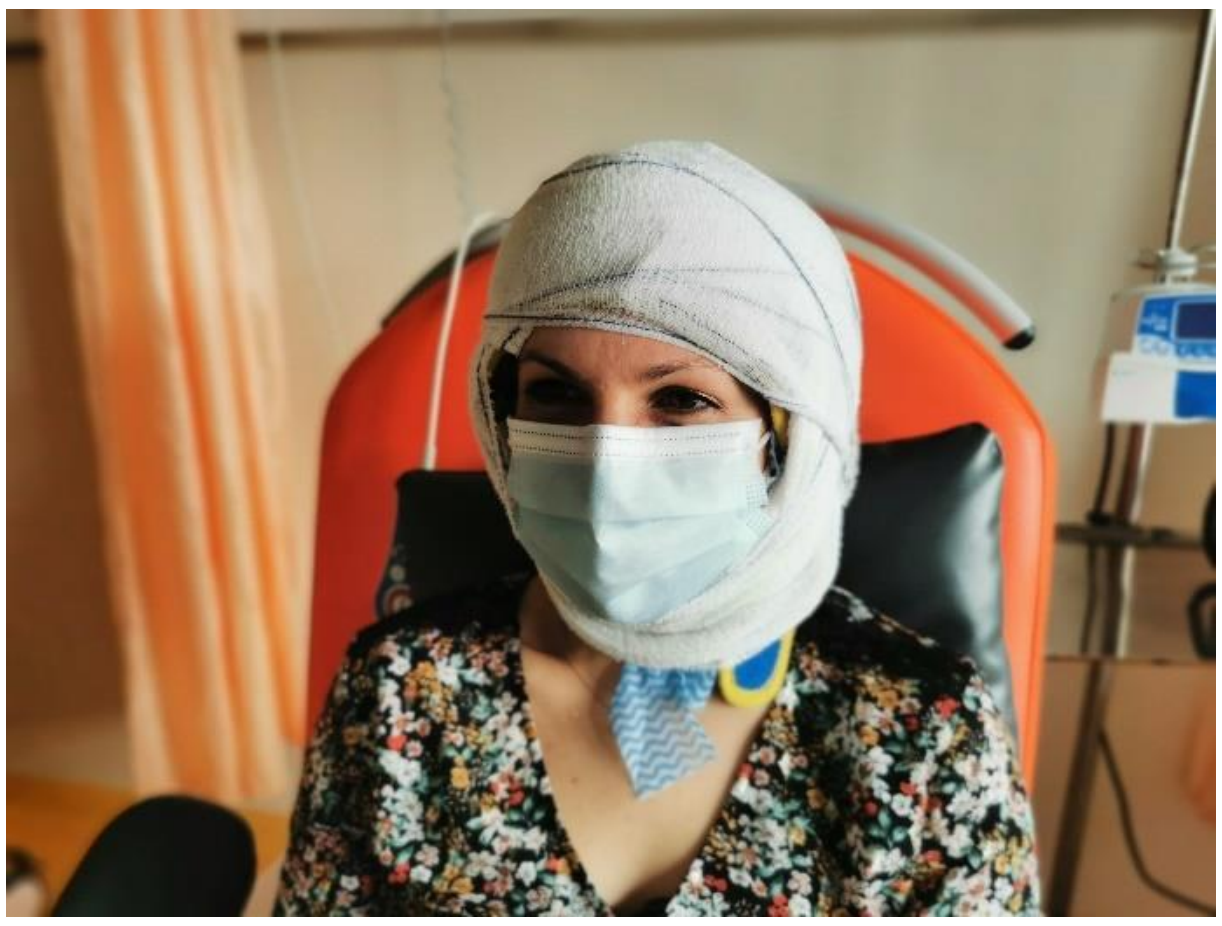

10.2 COOLING CAP BY SCALP COOLING: PAXMAN® PSCS 2 (EXPERIMENTAL GROUP)

10.2.1 Presentation

The scalp cooling cap used for patients assigned to the experimental group will be the PAXMAN® PSCS 2 cap, shown in the photo below. This cooling system circulates a cooling liquid at - 4°C through the cap's tubes to maintain a constant temperature across the entire scalp. The cooling tubes are supported by adjustable supports for maximum patient comfort and optimal fit. Therefore, it is not necessary to add a bandage to this device.

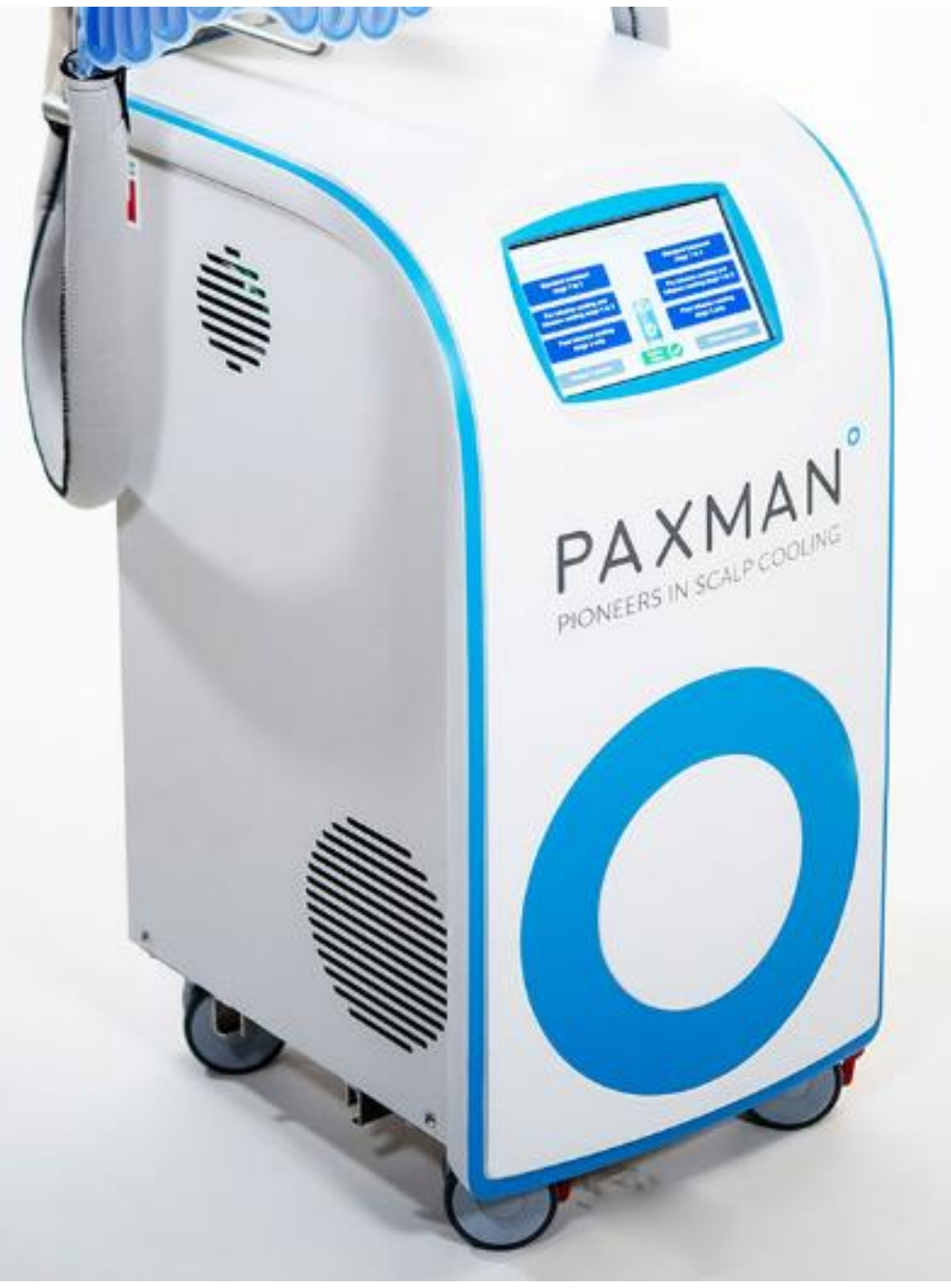

The cooling times recommended by the manufacturer depending on the molecules used are described in the table below:

Treatment	Minimal pre-cooling time	Infusion time	Minimal post-cooling time
EC	**30 minutes +**	Time required for drug infusion	**+ 120 minutes**
Daily Paclitaxel	**30 minutes +**	Time required for drug infusion	**+ 20 minutes**
Docetaxel	**30 minutes +**	Time required for drug infusion	**+ 20 minutes**
Thick or curly hair	**45 minutes +**	Duration of drug infusion	Duration as indicated above

The same cap will be kept in place for the duration of the treatment (no change of cap planned).

10.2.2 Setting up procedure

The scalp cooling technique will be used according to the following principles:

- Place a towel on the patient's shoulders.
- Lightly dampen the patient's hair with lukewarm water. If the patient has long hair, it is not necessary to wet the entire length, only the hair covering the scalp area. It is advisable to use a conditioner with a low alkaline or organic content.
- Apply a small amount of conditioner to damp hair.
- Comb the patient's damp hair back using a wide-toothed comb or your fingers so that the patient's frontal hairline is visible. This is particularly important for patients with bangs. If available, a stretchy mesh headband should be worn under each ear and across the forehead to create a barrier between the helmet and the patient's skin. This will reduce discomfort.

- Cap installation

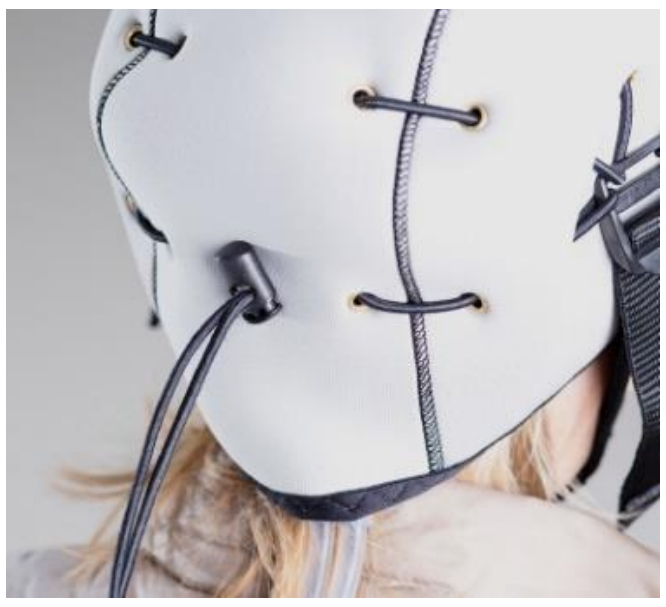

Configure the device according to the instructions provided by the manufacturer PAXMAN.

11 SAFETY

Vigilance will be conducted in accordance with current regulations. European Regulation No. 2017/745 on medical devices came into force on May 26, 2021, and requires the implementation of new measures concerning trial vigilance, including for trials authorized prior to that date. It introduces new definitions.

11.1 DEFINITIONS

11.1.1 Adverse Event (AE)

Any untoward medical occurrence, unintended disease or injury or any untoward clinical signs, including an abnormal laboratory finding, in subjects, users or other persons, in the context of a clinical investigation, whether or not related to the investigational device.

The nature of each adverse event, the date of occurrence, duration, severity, relationship to treatment, associated treatments, and outcome shall be established. The intensity of adverse events shall be rated according to criteria based on the condition being studied (CTC-NCIAE rating).

The investigator must specify whether the events are related to the research, the investigational products, associated medications, an underlying condition, or another cause.

11.1.2 Serious Adverse Events (SAE)

A *serious adverse event (SAE)* is defined as any adverse event that results in:

a) Death
b) A serious deterioration in the participant's state of health, which is the cause of:
    - A life-threatening illness or injury;
    - Permanent impairment of an anatomical structure or function;
    - Hospitalization or prolongation of the patient's hospitalization;
    - Medical or surgical intervention to prevent any illness or injury that endangers the patient's life or any permanent impairment of an anatomical structure or function;
    - Chronic illness;
c) Fetal distress, fetal death, congenital physical or mental impairments, or a congenital malformation

Certain “hospitalizations/extensions of hospitalization” are not considered serious adverse events:

- **Admission for social or administrative reasons**
- **Hospitalization predefined by the protocol**
- **Hospitalization for medical or surgical treatment scheduled before the research**
- **Hospitalization for pre-existing signs and symptoms that have not worsened**

It is therefore not necessary to notify them as SAEs.

Medical device defect (MD):
Any defect in the identity, quality, durability, reliability, safety, or performance of a medical device under investigation, including any malfunction, misuse, or defect in the information provided by the manufacturer.

New safety information:
Any new data that could lead to a reassessment of the benefits and risks of the research or the MD under study, to changes in the use of this MD, including the investigation procedure, in the conduct of the research, or in the documents relating to the research, or to the suspension, interruption, or modification of the research protocol or similar research.

11.2 INVESTIGATIOR RESPONSIBILITIES

11.2.1 Methods for detecting and collecting adverse events

All adverse events must be investigated, reported, recorded, treated, and evaluated.

Adverse events **occurring after the consent form has been signed will be recorded in the CRF for up to 8 days after the last application of the cooling cap**.

Each adverse event observed will be recorded individually. The severity of adverse events will be assessed according to the NCI-CTCAE version 5.0 classification (toxicity grades 1 to 5). The severity of adverse events not listed in this classification will be assessed according to the following criteria:

- Mild (grade 1): does not affect the patient's normal daily activities
- Moderate (grade 2): interferes with the patient's usual daily activities
- Severe (grade 3): prevents the patient's usual daily activities
- Very severe (grade 4): requires resuscitation measures/is life-threatening
- Death (grade 5): Death

The nature of each adverse event, its severity (*see section “SERIOUS adverse event or effect”*), date of onset/end, duration, severity, relationship to treatment, and progression will be established.

The investigator should specify whether the events are related to the research, the investigational products, associated medications, an underlying condition, disease progression, or another cause.

If an adverse event meets the definition of a serious advers e event, it must also be reported to the sponsor without delay using the dedicated form, in accordance with the procedures described in the section entitled “*Reporting of Serious Adverse Events*”.

11.2.2 Notification of Serious Adverse Events and Medical Device Defects

The investigator shall inform the Clinical Research Unit of all serious adverse events and medical device malfunctions that **occur during the study or within 8 days after the last application of the cooling cap**.

All delayed Serious Adverse Events (occurring after this 8-day period) considered reasonably related to the protocol treatment(s) or the research must be reported without delay.

The investigator must **notify the sponsor without delay (immediately) and no later than 3 calendar days after becoming aware** of any serious adverse events and device defects that occur during the trial, except for those listed in the protocol as not requiring immediate notification.

This notification shall be in the form of a written report and shall be followed, if necessary, by one or more detailed written follow-up reports.

The investigator shall note for each event/defect:

- ✓ Its description as clearly as possible using medical terminology
- ✓ Intensity
- ✓ Start and end date of the event
- ✓ The consequences of discontinuing use of the medical device and, where applicable, resuming its use,
- ✓ The measures taken and whether corrective treatment is necessary,
- ✓ Its progression. In the event of a non-fatal incident, its progression must be monitored until recovery or return to the previous state or stabilization of any sequelae.
- ✓ The **causal relationship** between this event and the **medical device** under investigation,
- ✓ The **causal relationship** between this event and the **procedure for implementing the MD, as well as the research procedures,**
- ✓ Relevant factors facilitating the assessment of the case, such as: associated conditions (which may be causally linked to the adverse event), medical and family history, results obtained after specific investigations.

To do so, they must use the dedicated study form provided in the Investigator's folder, which they must complete, date, and sign. They must also attach the laboratory results or examination or hospitalization reports detailing the serious event, including any relevant negative results, taking care to anonymize these documents so that only the patient's initials (first letter of surname and first letter of first name), gender, and date of birth (month/year) appear. Where applicable, they may also send the sponsor a copy of the autopsy report.

All these documents will be sent to the compliance department appointed by the sponsor, in accordance with the terms and conditions set out on the dedicated research form.
Clinical Research Unit, Pharmacovigilance Contact

R&D UNICANCER
Pharmacovigilance unit, France
Fax: +33 (0)1 44 23 55 70 or fax-pv@unicancer.fr
For any other communication:
Email: pv-rd@unicancer.fr / Phone: +33 (0)1 44 23 04 16

Additional information may be requested (by email, telephone, or during a visit) by the instructor or vigilant.
The investigator must document the event as thoroughly as possible, provide a **medical diagnosis** if possible, and establish a causal link between the serious adverse event and the research, the investigational products, associated medications, an underlying condition, disease progression, or another cause.
The investigator shall promptly communicate additional information concerning serious adverse events to the sponsor as it becomes known.
The investigator must follow up on patients who have experienced a serious adverse event **until resolution**, stabilization at a level deemed acceptable by the investigator, or return to the previous state, **even if the patient has withdrawn from the trial, and inform the sponsor of the progress of the serious adverse event.**

11.2.3 Special cases

11.2.3.1 Adverse Event of Special Interest (AESI)

Any serious or non-serious adverse event that requires special attention and will be specifically investigated. These AESIs must be reported and monitored in the same way as serious adverse events.

There are no AESIs in this study.

11.2.3.2 Serious Adverse Event that does not require immediate reporting

Any event that is part of the natural history of the disease (disease progression or hospitalization due to disease progression) should not be reported to pharmacovigilance on the SAE form but should be recorded in the research CRF.

Certain “hospitalizations/prolonged hospitalizations” are not considered serious adverse events and do not need to be reported to pharmacovigilance (see last paragraph, “*SERIOUS adverse event or effect*”).

11.3 SPONSOR RESPONSABILITY

11.3.1 Reference document for determining expected/unexpected events

It is the sponsor who determines whether an event is expected or unexpected in light of the reference document provided by the Sponsor.

11.3.2 Reporting of serious adverse events and device defects by the sponsor to the authorities

The sponsor states:

a) Any serious adverse event that has a proven or reasonably conceivable causal link with the MD under investigation, the comparator MD, or the investigation procedure;
b) Any defect in an MD that could have led to a serious adverse event in the absence of appropriate measures or intervention, or if circumstances had been less favorable;
c) Any new information concerning an event referred to in points (a) and (b).

11.3.3 New Facts

Any new facts relating to the research (or the product used) that could compromise the safety of the individuals participating in the research will be subject to appropriate urgent safety measures and immediate notification by the Sponsor to the competent authority and the Human Protection Committee.

12 STATISTICAL CONSIDERATIONS

12.1 SAMPLE SIZE

According to the literature, patients treated with chemotherapy for breast cancer following recommendations (3 EC and 3 Docetaxel, or 3 EC and 12 Paclitaxel) and using the standard cooling cap retain at least 50% of their hair in 20% to 36% of cases [Rice 2018; Katsimbri 2000].

The scalp cooling technique appears to be more effective in this indication, with up to 78% of patients retaining at least 50% of their hair after treatment with anthracyclines and taxanes [Gianotti, 2019].

The device (standard cooling cap or scalp cooling) will be considered effective if a CIA score of 0 (no hair loss) or a CIA score of 1 (less than 50% hair loss compared to the assessment before the start of chemotherapy) is observed at the end of chemotherapy, and ineffective if a grade 2 CIA is observed. Assuming an efficacy rate of 30% for the standard cooling cap and an efficacy rate of 50% for scalp cooling in preserving at least 50% of hair, it is necessary to observe 186 evaluable patients (93 per arm) to show a significant difference between the two arms, based on a two-sided proportion test with a 5% risk of type I error and 80% power.

In order to compensate for any non-evaluable patients, we increase this number by 5%, bringing the number of patients to be included to 196 (i.e., 98 per arm).

12.2 STATISTICAL ANALYSES

12.2.1 General considerations

Qualitative variables will be described using numbers and percentages, quantitative variables using the mean (± standard deviation) or the median and range if the normality assumption is not verified. The statistical significance threshold is set at 5% for each statistical analysis and confidence interval.

12.2.2 Main objective

The analysis of the primary endpoint will be performed on the intention-to-treat (ITT) population, i.e., considering the patient's randomization arm, regardless of the device actually received.
For the evaluation of the primary endpoint, the proportion of patients with grade 0 or 1 CIA at the end of chemotherapy treatment (anthracyclines and taxanes), assessed by centralized review of photographs of the scalp taken before and at the end of chemotherapy, will be estimated with a 95% two-sided confidence interval in each arm. It will be compared between the two arms using a two-sided Chi2 test.

12.2.3 Secondary objectives

Tolerance analysis:

The tolerance analysis population will correspond to patients who have used the helmet during at least one course of chemotherapy, regardless of whether the helmet was kept in place throughout the entire course of treatment.
The description of adverse events observed during the study will be carried out according to the following classifications:

- For each adverse event
- For each event attributable to the device
- For each event resulting in discontinuation of helmet use
- For each serious adverse event

Events will be distinguished according to when they occur: before treatment (symptoms present at inclusion), during treatment, and after treatment.
The frequency and number of patients who experience an adverse event will be described according to the maximum grade. The terminology used will be that of the MedDRA dictionary, allowing analyses to be performed by Preferred Term (PTNAME) and System Organ Classes (SOCNAME).

Analysis of subjective measures:

All questionnaire scores (OUTPATSAT 35, QLQ-C30, QLQ-BR23, HADS, and BIS) will be described by the mean and standard deviation in each arm, then compared using a Student t-test to assess the difference between the two arms at a fixed time point. The evolution of the measurements will be assessed by comparing the scores over time using a Student t-test for paired data in each arm. The score differences between inclusion and mid-treatment (before the first taxane treatment), and between inclusion and the end of chemotherapy, will be compared between the two arms using a Student t-test to measure any difference in changes between the arms.

13 MEDICAL-ECONOMIC EVALUATION

The medical-economic analysis will be conducted from the perspective of the community with a 12-month time horizon. The analysis population will be that of patients included in the trial, analyzed on an ITT basis.

We will conduct a cost-utility analysis, in which health outcomes will be measured in terms of survival and valued in QALYs, based on the assumption that scalp cooling will measurably improve patients' quality of life in the short term.
The EORTC QLQ C30 questionnaire will be administered to patients at inclusion, at the end of chemotherapy, and during the follow-up consultation with the oncologist. The different dimensions of the EORTC QLQ C30 questionnaire will be mapped to the dimensions of the EQ5D-5L questionnaire using the coefficients of an ordered logistic regression [Hagiwara, Y-2020].
We will then apply the French weightings to the dimensions of the EQ5D-5L questionnaire to obtain utilities [Andrade-2020] in each group. We will linearly interpolate the utility between each time point and obtain QALYs by calculating the area under the utility curve as a function of time ($dQALY/dt = S(t)U(t)$).

We will also perform a cost-effectiveness analysis using as the efficacy criterion the proportion of patients with a grade 0-1 CIA (according to NCI-CTCAE v.5.0 criteria) at the last chemotherapy treatment. Efficacy will be defined according to a binary composite criterion, 1 for a grade 0-1 CIA and 0 for any other CIA value.

The cost estimate will include all healthcare costs related to the monitoring and treatment of ACI throughout the trial. These costs will be discounted at an annual rate of 2.5%. Only direct costs will be included in the analysis, in accordance with the recommendations of the Haute Autorité de Santé (French National Authority for Health). The costs associated with the intervention will be collected:

- Purchase cost of the device
- Human resources cost: number of nurses and amount of work assigned to preparing for device insertion, monitoring, device removal, and cleaning, by performing micro-costing.

Our assumption is that hospitalizations are unlikely to occur as a result of using the device, as its benefits lie in comfort and quality of life. We therefore do not plan to collect data on hospital care consumption.
Human resources will be valued at the annual cost of the position, and the cost of the device will be valued at the hospital's central purchasing department.
We will calculate the cost-effectiveness ratios (ICER), defined as: (cost of standard care - cost of the scalp cooling strategy) / (QALY with standard care - QALY with scalp cooling).
Costs and utility will be presented as averages with 95% confidence intervals.

Finally, the robustness of the results will be assessed using deterministic sensitivity analysis to consider the uncertainty of the parameters studied by varying their values. A probabilistic analysis obtained by bootstrap will also be performed. The strategies will be compared in terms of cost-effectiveness. An acceptability curve will be produced to interpret the efficiency results according to the chosen threshold ratio.
Statistical analysis will be performed using the latest version of R® software.

14 TRIAL FOLLOW-UP

In order to guarantee the authenticity and credibility of the data in accordance with GCP, the sponsor will implement a quality assurance system that includes:

- Management of the trial in accordance with the procedures of the Clinical Research Unit,
- Quality control of investigator site data by the monitor, whose role is to verify

the consistency and coherence of the observation log data with respect to the source documents,

- The provision, if funding allows, of dedicated staff in the department to assist the investigator with study logistics and data collection in the observation logs.

15 ETHICS AND REGULATORY CONSIDERATIONS

The study is conducted in accordance with European Regulation (EU) 2017/745 on medical devices, the Bioethics Law, the law related to the protection of physical persons for the treatments of personal data and related to information technology, database and liberties, the Helsinki declaration, the Good Clinical Practices and ISO 14155 (GCP DM).

15.1 CLINICAL TRIAL APPROVALS

For this study within the scope of the clinical investigation regulation (IC 4.2), the sponsor must obtain to the French regulatory authorities before the study initiation:

- A favorable opinion of a Local Ethics Committee (Committee for the Protection of Persons, CPP)
- A validation by the Competent Health Authority (ANSM)

This study is under of the "Reference Methodology" (MR-001) in application of the provisions of article 54 paragraph 5 of the law of 6 January 1978 as amended relating to data processing, files and freedoms. This change was approved by decision of 5 January 2006.

The François Baclesse Center respects the regulations in force, in particular the rights of the persons being treated according to the EU regulation 2016/679 on the protection of data ("RGPD").

Any substantial modification in the protocol about objectives, design, population, evaluation, significant administrative modifications need the coordinator approval, the sponsor approval, CPP approval and if necessary the competent authority validation.

15.2 INFORMATION OF PATIENTS INVOLVED IN THE RESEARCH

Patients are completely and faithfully informed with understandable words on the objectives and constraints of the research, potential risks, required measures for monitoring and safety, of their right to decline the participation in the study or the possibility to withdraw from the study at any time.
All this information is included in the informed consents form given to the patient: one for the main study, other for ancillary studies. The investigator, or the physician who represents him, collects the signed written informed consent(s) before the definitive inclusion in the study. A copy of the information and consent form signed by the two parties is given to the patient; the investigator keeps the second copy.
For any significant modification of the protocol related to the objectives of the research, its design, the population, the exams or significant administrative aspects, a new consent from each person participating to the research is collected if needed.

15.3 PERSONAL DATA PROTECTION

All parties take all necessary actions required for the protection of subject personal data.

Each subject enrolled in the study is assigned a subject number and is referenced by this number and the subject's initials (1st letters of surname and first name). The data processing method is based on pseudonymization. Directly identifiable data is omitted from reports, publications, and other disclosures. All personal data are stored securely on the study site.

15.4 INVESTIGATOR RESPONSIBILITIES

The Principal Investigator of each participating center is committed to conduct the clinical trial in accordance with the trial protocol and with the regulations in force, notably the decision of 24 November 2006 related to Good Clinical Practices.
The Principal Investigator is responsible for:

- ✓ Giving to Sponsor his/her curriculum vitae and that of co-investigators
- ✓ Identifying persons involved in the research in his/her team and defining their responsibilities
- ✓ Initiating the inclusion of patients after Sponsor authorization
- ✓ Making the maximum effort to include the required number of patients within the established recruitment period.

Each Investigator is responsible for:

- ✓ Obtaining the signed and dated informed consent and personally signing this consent for each participating patient before any procedure specific to the trial
- ✓ Regularly completing the Case Report Form (CRF) for each patient included in the trial and to allow to CRAs mandated by the Sponsor a direct access to source data in order to validate the data entered in the CRF
- ✓ Dating, correcting and signing any correction in the CRFs and data clarification forms (DCF)
- ✓ Accepting the regular monitoring visits of the monitor and eventual auditors mandated by the Sponsor or inspectors of supervisory authorities

Source documents, defined as any document or original item that allows to prove the existence or accuracy of a data or a fact recorded during the study, are kept during 15 years by the investigator or the hospital if the source is a hospital medical record.

The archiving of the data is the responsibility of the investigator and according to the legislation. The patient should keep the data and a patient identification list for a minimum of 15 years after the end of the study.

16 DATA AND DOCUMENTS KEEPING

16.1 DATA ENTRY AND HANDLING

Data management is performed by the Data Processing Center (CTD) of the North West Cancéropôle (Centre de Traitement des Données du Cancéropôle Nord-Ouest). The CTD provides a database management software dedicated to clinical research: Ennov Clinical (version 8.2.50, ENNOV / CLINSIGHT, 33155 Cenon, France).

This software package, which is based on an Oracle database architecture, is designed for the overall management of clinical and epidemiological studies, meets the regulatory requirements related to this type of study. The CTD Ennov Clinical instance is validated in its computing environment. A data validation plan is developed jointly by the Clinical Research Unit and the Data Processing Center and describes in detail the controls to be performed for each variable.

A database specific to the study is created, tested and validated before the start of the study. All information required by the protocol must be recorded on the paper observation books - or on the electronic observation booklet - under the responsibility of the principal investigator and an explanation must be provided for each missing data item. The data have to be entered in these notebooks as they are obtained, and the sponsor takes over the monitoring.

The data are then checked by the CTD in accordance with the data validation plan. The database is frozen after final quality control and then exported to the adequate format for statistical analysis according to an automated and validated procedure.

16.2 ARCHIVING

The sponsor must ensure the archiving of essential documents on the conduct of the study in conditions ensuring their safety, for the minimum duration provided by GCP.

These documents are the protocol and annexes including any amendments, original signed information forms and consents, questionnaires, case report forms, follow-up documents, statistical analyses, the final report of the study.

The biological samples collected specifically for the biobank in this study are kept during 15 years.

16.3 PUBLICATION POLICY

The results of this study, property of the Sponsor (Centre François Baclesse), will be published under scientific articles. Publications relating to or resulting from this research will be communicated and submitted for review by the study coordinators to all investigators.

The authors include investigators that have included most patients, the biostatistician who has performed the data analysis, the clinical researcher monitor and the participants who provided substantial contribution to the development of the study, the analysis and interpretation of results and / or the writing of the manuscript.

17 FUNDING AND INSURANCE

17.1 FUNDING

Any additional costs referred to the Code of Public Health are being negotiated between the CFB and the representative of the institution, taking into account the financial resources available to the CFB in the frame of its public promotion activities.

However, the CFB ensures the study implementation and supply of the following material (protocol, CRF, investigator file) needed to the conduct of the study.

In the case of equipment or treatments are provided by other partners, the conditions must be specified in the study agreement.

17.2 INSURANCE

For the duration of the study, the sponsor has subscribed an insurance covering his own liability and that of any physician involved in the realization of the study. It also ensures full compensation for the harmful consequences to search for the person undergoing it and assigns, unless evidence against him that the damage is not attributable to its fault or that of any intervener, without that can be opposite the act of a third party or the voluntary withdrawal of the person who had originally agreed to participating to research. (Article L 1121-10).

18 REFERENCES

Andrade LF, Ludwig K, Goni JMR, Oppe M, de Pouvourville G. A French Value Set for the EQ-5D-5L. Pharmacoeconomics. 2020 Apr;38(4):413-425. doi: 10.1007/s40273-019-00876-4.PMID: 31912325; PMCID: PMC7080328.

Betticher DC, Delmore G, Breitenstein U, Anchisi S, Zimmerli-Schwab B, Müller A, von Moos R, Hügli-Dayer AM, Schefer H, Bodenmann S, Bühler V, Trueb RR. Efficacy and tolerability of two scalp cooling systems for the prevention of alopecia associated with docetaxel treatment. Support Care Cancer. 2013 Sep;21(9):2565-73. doi: 10.1007/s00520-013-1804-9. Epub 2013 May 2. PMID: 23636645.

Brédart, A., Swaine Verdier, A. & Dolbeault, S. Traduction/adaptation française de l'échelle "Body Image Scale" (BIS) évaluant la perception de l'image du corps chez des femmes atteintes de cancer du sein. Psycho Oncologie 1, 24–30 (2007). https://doi.org/10.1007/s11839-007-0001-9

Gianotti E, Razzini G, Bini M, Crivellaro C, Righi A, Darecchio S, Lui S, Basiricò ML, Cocconi S, Cagossi K, Ferrari A, Artioli F. Scalp Cooling in Daily Clinical Practice for Breast Cancer Patients Undergoing Curative Chemotherapy: A Multicenter Interventional Study. Asia Pac J Oncol Nurs. 2019 Jul-Sep;6(3):277-282. doi: 10.4103/apjon.apjon_4_19. PMID: 31259224; PMCID: PMC6518988.

Hagiwara Y, Shiroiwa T, Taira N, Kawahara T, Konomura K, Noto S, Fukuda T, Shimozuma K. Mapping EORTC QLQ-C30 and FACT-G onto EQ-5D-5L index for patients with cancer. Health Qual Life Outcomes. 2020 Nov 3;18(1):354. doi: 10.1186/s12955-020-01611-w. PMID: 33143687; PMCID: PMC7641825.

Hopwood P, Fletcher I, Lee A, et al. (2001) A body image scale for use with cancer patients. European Journal of Cancer 37: 189-97

Katsimbri P, Bamias A, Pavlidis N. Prevention of chemotherapy-induced alopecia using an effective scalp cooling system. Eur J Cancer. 2000 Apr;36(6):766-71. doi: 10.1016/s0959-8049(00)00012-5. PMID: 10762750

Lemieux J, Maunsell E, Provencher L. Chemotherapy-induced alopecia and effects on quality of life among women with breast cancer: a literature review. Psychooncology. 2008 Apr;17(4):317-28. doi: 10.1002/pon.1245. PMID: 17721909.

Martín M, de la Torre-Montero JC, López-Tarruella S, Pinilla K, Casado A, Fernandez S, Jerez Y, Puente J, Palomero I, González Del Val R, Del Monte-Millan M, Massarrah T, Vila C, García Paredes B, García-Sáenz JA, Lluch A. Persistent major alopecia following adjuvant docetaxel for breast cancer: incidence, characteristics, and prevention with scalp cooling. Breast Cancer Res Treat. 2018 Oct;171(3):627-634. doi: 10.1007/s10549-018-4855-2. Epub 2018 Jun 19. Erratum in: Breast Cancer Res Treat. 2018 Jul 16;: PMID: 29923063; PMCID: PMC6133184.Protocole ICELAND N°ID-RCB : 2023-A00769-36 - Version n°4.1 du 26/05/2025 Page 36 sur 36

Rugo HS, Melin SA, Voigt J. Scalp cooling with adjuvant/neoadjuvant chemotherapy for breast cancer and the risk of scalp metastases: systematic review and meta-analysis. Breast Cancer Res Treat. 2017 Jun;163(2):199-205. doi: 10.1007/s10549-017-4185-9. Epub 2017 Mar 8. PMID: 28275922; PMCID: PMC5410200.

Rugo HS, Voigt J. Scalp Hypothermia for Preventing Alopecia During Chemotherapy. A Systematic Review and Meta-Analysis of Randomized Controlled Trials. Clin Breast Cancer. 2018 Feb;18(1):19-28. doi: 10.1016/j.clbc.2017.07.012. Epub 2017 Aug 10. PMID: 28939291.

Rice BA, Ver Hoeve ES, DeLuca AN, Esserman LJ, Rugo HS, Melisko ME. Registry study to assess hair loss prevention with the Penguin Cold Cap in breast cancer patients receiving chemotherapy. Breast Cancer Res Treat. 2018 Jan;167(1):117-122. doi: 10.1007/s10549-017-4506-z. Epub 2017 Sep 18. PMID: 28921303.

Zhou T, Han S, Zhu Z, Hu Y, Xing W. Interventions for Preventing Chemotherapy-Induced Alopecia: A Systematic Review and Network Meta-analysis of Randomized Controlled Trials. Cancer Nurs. 2021 Nov-Dec 01;44(6):E567-E577. doi: 10.1097/NCC.0000000000000899. PMID: 33003120
